# A Multifunctional Photothermal Catalyst Enabling Full‐Day Sustainable Power and Indoor Air Quality Control

**DOI:** 10.1002/advs.202505059

**Published:** 2025-06-20

**Authors:** Niansi Li, Wei Wei, Yulin Li, Feiyang Xu, Guoyu Zhang, Jie Ji, Xudong Zhao, Junwei Liu, Bendong Yu, Qiliang Wang

**Affiliations:** ^1^ College of Urban Construction Nanjing Tech University Nanjing Jiangsu 210009 China; ^2^ Department of Thermal Science and Energy Engineering University of Science and Technology of China Hefei China 230026; ^3^ Centre for Sustainable Energy Technologies University of Hull Hull HU6 7RX UK; ^4^ Department of Building Environment and Energy Engineering The Hong Kong Polytechnic University Kowloon Hong Kong 999077; ^5^ Department of Architecture and Built Environment University of Nottingham University Park Nottingham NG7 2RD UK

**Keywords:** dehumidification, framework materials, metal–organic, photothermal catalysis, photothermal catalytic energy utilization, thermoelectric power generation

## Abstract

Solar photothermal catalytic (PTC) purification holds great potential for indoor air pollution control, but efficiently collecting energy at the catalytic interface to maximize solar energy utilization and meet building requirements remains a global challenge. This study develops a novel infrared‐light‐driven photothermal catalyst, Mn_7_Co_3_Ce_1_O_x_, which efficiently utilizes solar and ambient energy, offering three functions: catalysis, heating, and cooling. Furthermore, this study proposes an innovative hybrid system that integrates PTC film, thermoelectric generator (TEG), and metal–organic frameworks (MOFs). Through synergetic effects, the system combines the heating‐catalysis‐cooling effect of the PTC film with the moisture‐induced adsorption/desorption heat of the MOFs, achieving year‐round power generation, dehumidification, and air purification. Results show that Mn_7_Co_3_Ce_1_O_x_ has a full‐spectrum solar absorptivity of 94.3% and an atmospheric window emissivity of 95.7%. Under infrared light, the pollutant removal rate on the photothermal catalytic interface reaches 90.9%. Within 4 h, the thermoelectric power density increased by 640.1%, the relative humidity decreased by 16.8%, and the clean air volume reached 270.8 m^3^·h^−1^·m^−2^. When the system is applied to building rooftops in 34 provincial capital cities in China, the simulation results show annual power density of 30–105.3 kW·m⁻^2^, air conditioning energy savings of 11.2–353.5 kW·m^−2^, and clean air volume of 296.3–1119.3 m^3^·h^−1^·m^−2^.

## Graphical abstract

In this study, a novel photothermal catalyst, Mn_7_Co_3_Ce_1_O_x_ has been developed, which features a type‐II heterojunction structure that enables broad‐spectrum absorption, including visible and infrared light. This material significantly enhances the photothermal catalytic efficiency by overcoming the limitations of traditional catalysts that struggle to absorb the full solar spectrum. Additionally, the catalyst demonstrates excellent radiative cooling properties, effectively reducing temperature during the night and improving overall energy efficiency. Building upon this advanced material, an innovative hybrid system, the PTC‐TEG‐MOF system is designed, which integrates photothermal catalysis, thermoelectric generation (TEG), and radiative cooling to offer a comprehensive solution for indoor air purification, energy harvesting, and temperature regulation in all weather conditions. This system provides a sustainable approach to improving indoor air quality while enhancing energy efficiency.

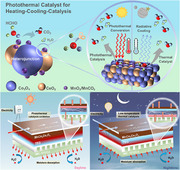

## 1 Introduction

People spend more than 90% of their time indoors, making a healthy and comfortable indoor environment especially important for human health.^[^ ^1^ ^]^ Air conditioning systems play an important role in indoor environment conditioning, along with one of the major carbon emissions in the building sector.^[^ ^2^ ^]^ Sufficient outdoor fresh air is needed to be introduced to dilute indoor pollutants such as volatile organic compounds (VOCs[3]) with energy consumption up to 35–40 kWh·m^−2^·year^−1^. Moreover, the outdoor fresh air is no longer “fresh” when outdoor air is polluted.^[^ ^4^, ^5^ ^]^ Meanwhile, the energy consumption for indoor humidity treatment accounts for ≈30% of total air‐conditioning systems. Therefore, the efficiently removal on indoor pollutants and humidity by low energy consumption is a key challenge to achieving building carbon reduction.^[^ ^6^, ^7^ ^]^

Active indoor air purification technology can effectively reduce indoor pollutant concentration without increasing air conditioning loads.^[^ ^8^, ^9^ ^]^ Mian air purification technologies such as filtration, adsorption, photothermal catalysis, and plasma oxidation have attracted widespread attention from scholars worldwide.^[^ ^10^ ^]^ During these processes, photothermal catalysis generates free radicals by absorbing the high‐energy portion of sunlight, degrading pollutants into CO_2_ and H_2_O.^[^ ^11^, ^12^ ^]^ At the same time, it converts the remaining low‐energy light into thermal energy, thereby enhancing the synergistic effect of light and heat.^[^ ^13^, ^14^ ^]^ Based on the light response range, photothermal catalysts can be classified into three types: ultraviolet light (UV) driven, visible light (Vis) driven, and near‐infrared light (IF) driven type.^[^ ^15^, ^16^ ^]^ Common photothermal catalytic materials include metal oxides such as TiO_2_^[^ ^17^ ^]^ and iron oxides,^[^ ^18^ ^]^ carbon‐based materials such as graphene^[^ ^19^ ^]^ and carbon nanotubes,^[^ ^20^ ^]^ and metal composite materials such as copper‐titanium composites.^[^ ^21^ ^]^ Given the limited sunlight received by building surfaces, developing photothermal catalysts with high solar full‐spectrum absorptivity, particularly those capable of utilizing long‐wavelength light (such as such as Vis or IR), has become a significant challenge.^[^ ^22^ ^]^

To broaden the light response range of photothermal catalysts, various methods have been employed, such as bandgap engineering, surface modification, and nanostructure regulation.^[^ ^23^, ^24^, ^25^ ^]^ Common strategies including doping, constructing heterojunctions, introducing noble metal nanoparticles, and incorporating photosensitive dyes.^[^ ^23^, ^26^, ^27^ ^]^ These approaches significantly enhance photothermal catalytic performance through synergistic effects, tunable electronic properties, high selectivity, and durability.^[^ ^28^ ^]^ Among these strategies, surface modification, doping, and nanostructure design are commonly employed in material design to achieve specific light‐response bands, primarily in the visible light range.^[^ ^29^, ^30^, ^31^ ^]^ Composite metal oxide heterojunctions, formed by combining materials with different bandgaps, can absorb light over a broader wavelength range, particularly in the visible and near‐infrared regions.^[^ ^32^, ^33^, ^34^ ^]^ Additionally, the interface effects of composite heterojunctions generate effective interfacial electric fields that facilitate electron transfer and carrier separation, thereby enhancing carrier separation efficiency across a wider range and improving photothermal catalytic performance.^[^ ^35^, ^36^ ^]^ Herein, a novel photothermal catalyst Mn_7_Co_3_Ce_1_O*_x_* with type‐II heterojunction is developed. The catalyst has a high full solar spectrum absorptivity of 94.3%, with a fast temperature increase of up to 68 °C under 1000 W m^−2^. The photocatalytic light response range is efficiently extended to 1600 nm, achieving an optimal photothermal catalytic performance of 95.4%. Surprisingly, the catalyst has a high solar emissivity of 95.7% in the atmospheric window, achieving a cooling temperature difference of 5 °C under a high‐temperature nighttime environment. This photothermal catalyst with heating‐cooling‐catalysis functions, showcases huge application potential for air purification and thermal regulation in buildings.

Furthermore, we have innovatively proposed an efficient energy collection strategy by integrating the photothermal catalytic interface with solid‐state dehumidifying metal–organic frameworks (MOFs) and thermoelectric generators (TEGs), forming the PTC‐TEG‐MOF hybrid system. This system leverages the Mn_7_Co_3_Ce_1_O*_x_* interface to achieve multiple functions, including air purification, power generation, and dehumidification. During the daytime, sunlight drives the photothermal catalytic oxidation reaction and moisture desorption of MOFs, achieving air purification and moisture regeneration; at night, the low‐temperature thermocatalytic effect combined with MOF moisture absorption synergistically achieves air purification and dehumidification. TEG generates 24 h power by utilizing the temperature difference between the MOF and photothermal catalysts, significantly enhancing the system's power generation, dehumidification, and purification performance.

Unlike traditional systems that focus solely on power generation or air purification,^[^ ^37^, ^38^, ^39^ ^]^ this system recovers waste heat to achieve continuous 24 h power generation and dehumidification, significantly improving energy efficiency and overall system performance. The Mn_7_Co_3_Ce_1_O*_x_* photothermal catalytic material exhibits excellent catalytic performance under visible light and enhances radiative cooling in the atmospheric window, meeting the energy conversion needs in building environments. Compared to previous studies,^[^ ^40^, ^41^, ^42^, ^43^, ^44^ ^]^ this material maintains high purification efficiency even after multiple uses, demonstrating greater stability. Furthermore, the system significantly outperforms existing research in key performance indicators such as purification efficiency and power generation, providing a highly efficient and sustainable building energy solution, particularly suitable for regions with low light or large temperature variations.

When the hybrid system is applied to the rooftops of buildings across China, it demonstrates significant annual benefits, including air purification, air conditioning energy savings, and power generation. Simulation results show an annual electrical power density range of 30–105.3 kW·m^−2^, annual air conditioning energy savings of 11.2 – 353.5 kW·m^−2^, and an annual clean air volume of 296.3 – 1119.3 m^3^·h^−1^·m^−2^. Our research achieves simultaneous power generation, dehumidification, and purification, providing a promising pathway for building decarbonization.

## 2 Results

### 2.1 Synthesis and Characterizations of Photothermal Catalyst for Heating‐Cooling‐Catalysis

**Figure 1:**
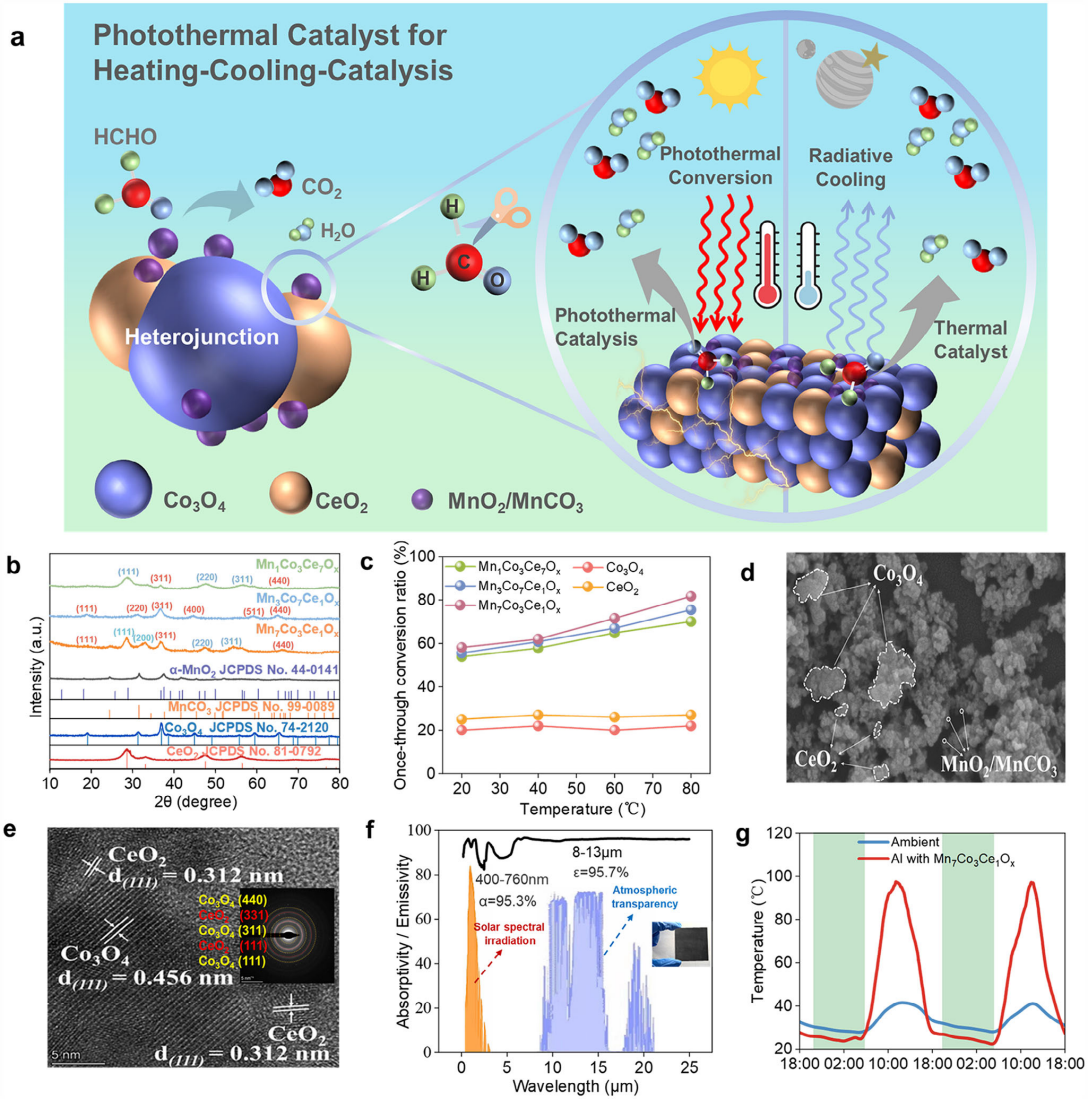
The characteristic of photothermal catalytic materials. a) Schematic diagram of photothermal catalyst for heating‐cooling‐catalysis. b) The XRD images of the photothermal catalyst samples with various molar ratios. c) The once‐through conversion ratio of the samples. d) The SEM images of Mn_7_Co_3_Ce_1_O*_x_*. e) The TEM images of Mn_7_Co_3_Ce_1_O*_x_*. f) The full‐spectrum characteristics of Mn_7_Co_3_Ce_1_O*_x_*. g Outdoor experimental tests on the surface temperature of Mn_7_Co_3_Ce_1_O*_x_*.

A novel photothermal catalyst with a type‐II heterojunction has been successfully developed (**Figure** **1**a), demonstrating outstanding performance in photothermal conversion and catalytic processes. The catalyst was synthesized via a co‐precipitation method, using cerium nitrate, cobalt nitrate, and manganese acetate as precursors, followed by heat treatment at 400 °C to obtain the sample (Figure S1, Supporting Information), exhibiting excellent performance in various catalytic applications. In tests with different molar ratios, the Mn_7_Co_3_Ce_1_O*_x_* sample with a molar ratio of 7:3:1 achieved the highest single‐pass conversion efficiency of 81.7% at 80 °C (Figure 1c), demonstrating the optimal composition of the catalytic material. XRD analysis confirmed that Co_3_O_4_ (JCPDS No. 74–2120) and CeO_2_ (JCPDS No. 81–0792) are the major crystalline phases, while MnO_2_/MnCO_3_ (JCPDS No. 44–0141, JCPDS No. 9‐0089) are uniformly dispersed in the Co_3_O_4_ and CeO_2_ matrix as weakly crystalline ultrafine nanoparticles (Figure 1b). Scanning electron microscopy (SEM) images show that Co_3_O_4_ forms aggregated nanoparticles in the range of 50–300 nm, while CeO_2_ aggregates have sizes between 50 and 100 nm, and the MnO_2_/MnCO_3_ nanoparticles are uniformly distributed (Figure 1d).

SEM and transmission electron microscopy (TEM) analyses confirmed the heterostructure with CeO_2_ (111) and amorphous MnO_2_/MnCO_3_ stacked on Co_3_O_4_ (111) (Figure 1e). This unique structure contributes to efficient solar light absorption and facilitates the activation of oxygen molecules, which is crucial for enhancing the photothermal catalytic efficiency.^[^ ^45^, ^46^ ^]^ As depicted, the EPR signal at g ≈ 2.003 commonly associated with oxygen is significantly enhanced in Mn_7_Co_3_Ce_1_O_x_. EPR patterns indicate that the ternary composite possesses the highest oxygen vacancy concentration (Figure S2, Supporting Information). Spectral analysis shows that Mn_7_Co_3_Ce_1_O*_x_* exhibits excellent absorptivity in the visible spectrum (400–760 nm), with an average of 95.3%, and a high emissivity in the atmospheric window (8–13 µm), with an average of 95.7% (Figure 1f). These optical properties make the catalyst highly promising for applications in solar thermal collection, purification, and radiative cooling.Outdoor experimental tests show that the Mn_7_Co_3_Ce_1_O*_x_* catalyst can increase the surface temperature by up to 66.1 °C during the day, with a temperature drop of 4–5 °C at night (Figure 1g). The combination of photothermal conversion efficiency, excellent thermal management properties, and unique design makes Mn_7_Co_3_Ce_1_O*_x_* a promising candidate for sustainable energy applications, capable of achieving purification, heating, and cooling functions in a single material.

**Figure 2:**
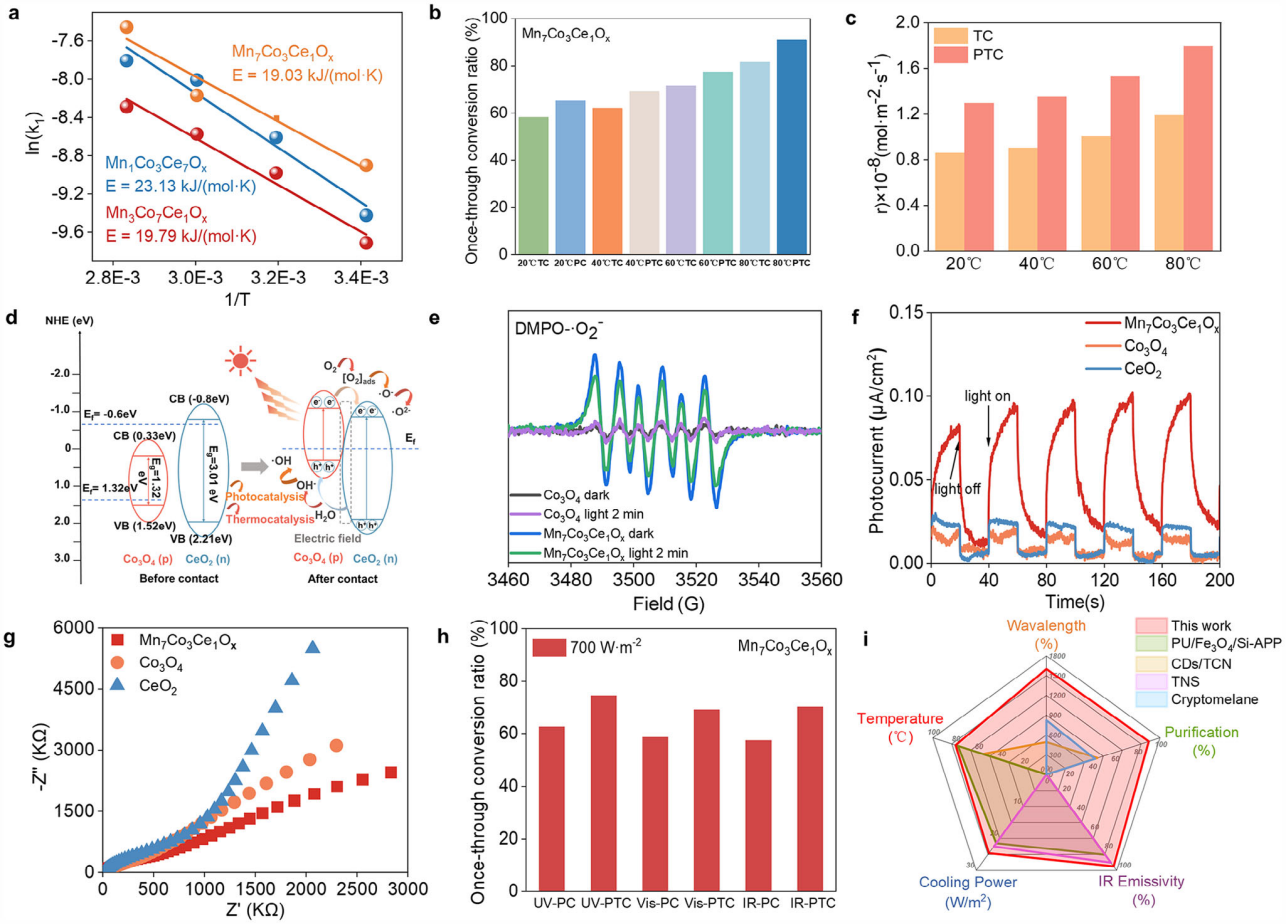
The comprehensive performance of Mn_7_Co_3_Ce_1_O*_x_*. a) The L‐H bimolecular dynamics simulation and activation energy. b) The single‐pass ratio of Mn_7_Co_3_Ce_1_O_x_ under photothermal coupling catalysis conditions. c) The reaction rate of Mn_7_Co_3_Ce_1_O*_x_*. d) The energy band structure distribution diagram of Mn_7_Co_3_Ce_1_O*_x_*. e) The characteristic signal of Mn_7_Co_3_Ce_1_O_x_ superoxide anion radical in a dark environment. f) The transient photocurrent response test of Mn_7_Co_3_Ce_1_O*_x_*. g) The resistance evolution of Mn_7_Co_3_Ce_1_O*_x_*, Co_3_O_4,_ and CeO_2_. h) The single‐pass ratio of Mn_7_Co_3_Ce_1_O*_x_* under different optical bands. i) The comparison of Mn_7_Co_3_Ce_1_O*_x_* with other materials.

**Figure** **2** illustrates the catalytic performance of Mn_7_Co_3_Ce_1_O*_x_* under thermal and photo‐thermal conditions. Kinetic analysis based on the Langmuir–Hinshelwood model reveals an activation energy of 19.03 kJ·mol^−1^·K^−1^, which is lower than that of Mn_1_Co_3_Ce_7_O*_x_* (23.13) and Mn_3_Co_7_Ce_1_O*_x_* (19.79) (Figure 2a). This indicates that the optimized composition offers improved thermodynamic properties, enabling efficient catalysis at lower temperatures. Under identical conditions (80 °C, 100 ppb HCHO, 1000 W·m^−2^), the formaldehyde single‐pass conversion rate reached 90.85% in the photo‐thermal system, exceeding that of thermal‐only catalysis (81.69%) by 9.16% (Figure 2b). This confirms the synergistic enhancement effect of photo‐thermal coupling. Arrhenius plots suggest that photothermal‐coupled catalysts leverage the solar spectrum to achieve enhanced degradation of gaseous formaldehyde through a synergistic photothermal effect (Figure S3 and Table S3, Supporting Information). In terms of reaction rate, the peak rate under photo‐thermal conditions reached 1.81 mol·m^−2^·s^−1^, consistently outperforming thermal catalysis across the entire temperature range (Figure 2c). Additionally, CO_2_ selectivity increased from 58.14% to 81.69% as the temperature raised from 20 to 80 °C, with no detectable byproducts observed (Figure S5, Supporting Information). The catalyst also maintains excellent stability after 100 reaction cycles (Figure S4, Supporting Information).

The underlying mechanism involves a type‐II heterojunction formed between CeO_2_ and Co_3_O_4_, which facilitates efficient charge separation under the built‐in electric field (Figure 2d). Photo‐generated electrons transfer from the conduction band of Co_3_O_4_ to CeO_2_, while holes move in the opposite direction, promoting spatial charge separation at the interface. Combined with XRD, which confirmed phase coexistence, the EDS mapping further supported the formation of a heterojunction structure, which facilitated electron transfer across the heterointerface (Figure S5, Supporting Information). ESR spectroscopy shows a significantly enhanced ·O_2_⁻ signal under light irradiation (Figure 2e), suggesting more active oxygen species are generated due to more effective electron transfer. Transient photocurrent measurements further confirm that Mn_3_Co_7_Ce_1_O*_x_* exhibits much stronger photocurrent responses than individual CeO_2_ or Co_3_O_4_ (Figure 2f), indicating superior charge separation and transfer efficiency. Electrochemical impedance spectroscopy (EIS) shows the smallest Nyquist arc radius for Mn_3_Co_7_Ce_1_O*_x_* (Figure 2g), reflecting lower interfacial resistance and better charge transport characteristics. To investigate the effect of light wavelength on purification performance, conversion rates were measured under UV, visible, and near‐infrared light at 700 W m^−2^ and 300 ppb formaldehyde. UV light delivers the highest single‐pass conversion rate (74.5%), followed by near‐infrared (70.21%) and visible light (69.13%) (Figure 2h). Based on Planck's formula, the excitation wavelength of Mn_7_Co_3_Ce_1_O_x_ was calculated to reach 1600 nm (Figure S6, Supporting Information). Finally, a comprehensive comparison with previously reported catalysts shows that Mn_3_Co_7_Ce_1_O*_x_* exhibits the best overall performance in terms of spectral response, purification efficiency, infrared emissivity, cooling power, and working temperature (Figure 2i).^[^ ^40^, ^41^, ^42^, ^43^ ^]^

### 2.2 Principle and Proof‐Of‐Concept of Hybrid PTC‐TEG‐MOF Device

**Figure 3:**
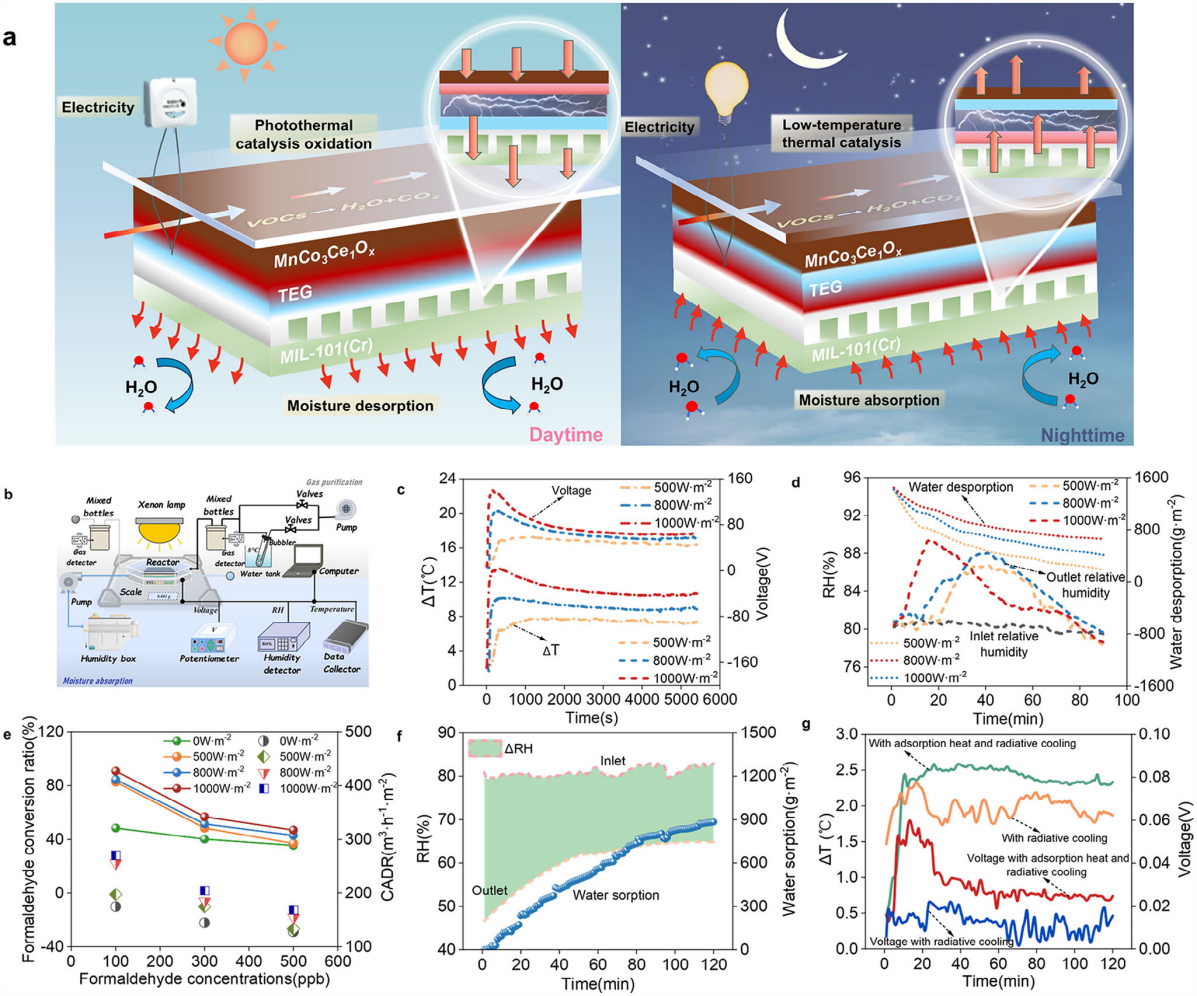
The all‐day air purification, power generation, and air dehumidification device enabled by energy harvesting on the photothermal catalytic interface integrated with MOF and TEG. a) The operation principle of the hybrid device during the daytime and nighttime. b) The experimental tests on the air purification, power generation, and air dehumidification performance in the laboratory. c) The temperature difference and voltage of TEG under different sunlight intensities. d) The air relative humidity and water desorption rate of MOF under different sunlight intensities. e) The formaldehyde conversion ratio and CADR of the hybrid device under different sunlight intensities. f) The air relative humidity and water sorption of MOF during the dehumidification process. g) The temperature difference and voltage of TEG in the devices with and without MOF. All experiments were conducted at a typical ambient condition of 28 °C and 80% relative humidity.

Building on the Mn_3_Co_7_Ce_1_O*_x_* photo‐thermal catalytic material, we further fabricated an infrared‐responsive photo‐thermal catalytic film (PTC film). This film exhibits excellent solar absorption, photo‐thermal catalytic efficiency, and radiative cooling capability. To fully harness its energy conversion potential under all‐weather conditions, we designed a self‐powered multifunctional device. It integrates air purification, power generation, and humidity control via a photo‐thermal catalytic interface, which we termed the PTC‐TEG‐MOF hybrid system (**Figure** **3**a). The device comprises three functional modules: the PTC film, thermoelectric generator (TEG), and metal–organic frameworks (MOFs)‐based solid‐state dehumidifier. These components are assembled sequentially using high‐thermal‐conductivity adhesive to ensure efficient heat transfer. The PTC film serves as the core multifunctional layer, responsible for VOC degradation, solar‐to‐thermal conversion, and passive radiative cooling. The MOF module operates via cyclic adsorption–desorption to achieve daytime regeneration and nighttime dehumidification. The TEG module exploits the temperature gradient between the PTC and MOF layers to continuously convert thermal energy intoelectricity.

To address all‐weather dehumidification and optimize thermal management, MOFs were introduced as solid‐state desiccants. MOFs such as MIL‐101(Cr),^[^ ^47^, ^48^, ^49^, ^50^ ^]^ MOF‐801,^[^ ^51^, ^52^ ^]^ and MIL‐100(Fe) ^[^ ^53^ ^]^ have attracted considerable attention due to their eco‐friendliness, high surface area, tunable pore structure, and superior moisture adsorption capacity. In this study, MIL‐101(Cr) was selected as the desiccant material due to its high‐water uptake and rapid adsorption kinetics. Moreover, its proven performance under real atmospheric conditions makes it a suitable candidate for both outdoor and indoor dehumidification applications.^[^ ^54^ ^]^ Meanwhile, considering the low thermal conductivity of MIL‐101(Cr), we prepared a MIL‐101(Cr)/copper foam (CF) composite as the desiccant by coating MIL‐101(Cr) powder onto copper foam via an in‐situ impregnation method. Water adsorption isotherms reveal that MIL‐101(Cr)/CF exhibits fast and efficient water uptake at 20 °C (Figure S7, Supporting Information). Its water adsorption performance under various temperatures and relative humidity levels demonstrates strong environmental adaptability (Figure S8, Supporting Information). Furthermore, cyclic adsorption–desorption tests show a stable water uptake capacity of approximately 0.925 g·g^−1^, indicating excellent long‐term cycling stability (Figure S9, Supporting Information). The adsorption‐desorption cycles results show that its adsorption capacity remains between 0.87 and 0.93 g·g⁻^1^, with a performance decay of less than 5% after 100 cycles, demonstrating excellent stability (Figure S10, Supporting Information).

Daytime Mode: Under sunlight, the PTC film absorbs broadband solar energy (<1600 nm), enabling photo‐thermal catalytic degradation of indoor volatile organic compounds (VOCs) into CO_2_ and H_2_O. Simultaneously, thermal siphon effects facilitate indoor air circulation. The absorbed solar heat is transferred to the TEG and MOF layers. Heat triggers water desorption in the MOF, regenerating the material, while the TEG module converts the resulting temperature gradient into continuous power output.

Nighttime Mode: In the absence of sunlight, the PTC film functions as a low‐temperature catalytic platform. Its surface temperature decreases due to radiative cooling, allowing continued VOC degradation. The MOF module adsorbs atmospheric moisture at low temperature, completing the dehumidification cycle and releasing adsorption heat. This thermal energy elevates the hot‐side temperature of the TEG, enabling it to maintain power generation throughout the night.

This system enables full‐spectrum solar utilization (visible–NIR) and integrates air purification, energy harvesting, and humidity regulation into a single platform, demonstrating substantial application potential. Notably, this work reports for the first time a heating–cooling–catalysis synergy based on a photo‐thermal interface. This integrated system enables 24 h electricity generation, dehumidification, and indoor air purification without any energy storage.

To experimentally validate the concept of continuous energy harvesting and multifunctional application via a photothermal‐catalytic interface, we designed and assembled a hybrid PTC‐TEG‐MOF device. This integrated system enables day‐night cycle operation for air purification, thermoelectric power generation, and humidity regulation.

Daytime operation: Under simulated solar irradiation at intensities of 500, 800, and 1000 W·m^−2^, the surface temperature of the Mn_3_Co_7_Ce_1_O*_x_*‐based PTC film rapidly increased to 52, 60, and 68 °C, respectively (Figure S11, Supporting Information). These results demonstrate its excellent solar‐thermal conversion capability. The corresponding temperature differences (ΔT) across the TEG module reached 7.8 °C, 10.2 °C, and 13.6 °C, resulting in open‐circuit voltages (*Voc*) of 58.7, 105, and 141 mV, respectively (Figure 3c). The maximum power densities achieved were 0.10, 0.32, and 0.57 W·m^−2^ (Figure S12, Supporting Information), with associated conversion efficiencies of 0.17%, 0.24%, and 0.35% (Figure S13, Supporting Information). Meanwhile, photothermal catalysis triggered by NIR light (λ < 1600 nm) enabled efficient VOC degradation. As formaldehyde (HCHO) concentration increased, both the removal efficiency and clean air delivery rate (*CADR*) decreased. However, under a low inlet concentration of 100 ppb and a flow rate of 5 L·min^−1^, the formaldehyde removal efficiency still reached 90.9%, with a CADR as high as 270.8 m^3^·h^−1^·m^−2^ (Figure 3e). Concurrently, the solar heat input accelerated moisture desorption from MIL‐101(Cr) (Figure S14, Supporting Information), achieving a water desorption rate of 698 g·m^−2^·h^−1^ under 1000 W·m^−2^ (Figure 3d), and effectively realizing a thermosiphon effect for indoor air circulation (Figure S15, Supporting Information).

Nighttime operation: In simulated nighttime conditions (T = 28 °C, RH = 80%), the device demonstrated a different working mode driven by radiative cooling and low‐temperature thermal catalysis. The MIL‐101(Cr)@CF MOF layer effectively adsorbed ambient moisture, with a total adsorption capacity of 533.57 g·m^−2^ and an average rate of 276.79 g·m^−2^·h^−1^ (Figure 3f). This reduced the ambient relative humidity from 80% to 45–60% within 2 h, aligning with the ASHRAE recommended indoor RH range (40%–65%) (Figure 3g).

To evaluate the thermoelectric enhancement by MOF's adsorption heat, we compared the hybrid device to a reference PTC‐TEG‐Air module without the MOF layer. During adsorption, the exothermic process caused a temperature rise of 3.2 °C at the hot end in the initial stage and maintained a 2.45 °C increase over time (Figure 3f, Figure S16, Supporting Information). Consequently, the hybrid PTC‐TEG‐MOF module achieved a maximum *Voc* of 61.7 mV—approximately 2.7 higher than the 22.7 mV of the reference PTC‐TEG‐Air module (Figure 3g). The peak power density reached 108.8 mW·m^−2^, marking a 640.14% increase over the reference device (14.7 mW·m^−2^, Figure S17, Supporting Information), underscoring the significant contribution of MOF adsorption heat as a nighttime thermal energy source.

### 2.3 Outdoor Demonstration of Air Purification, Power Generation, and Dehumidification

**Figure 4:**
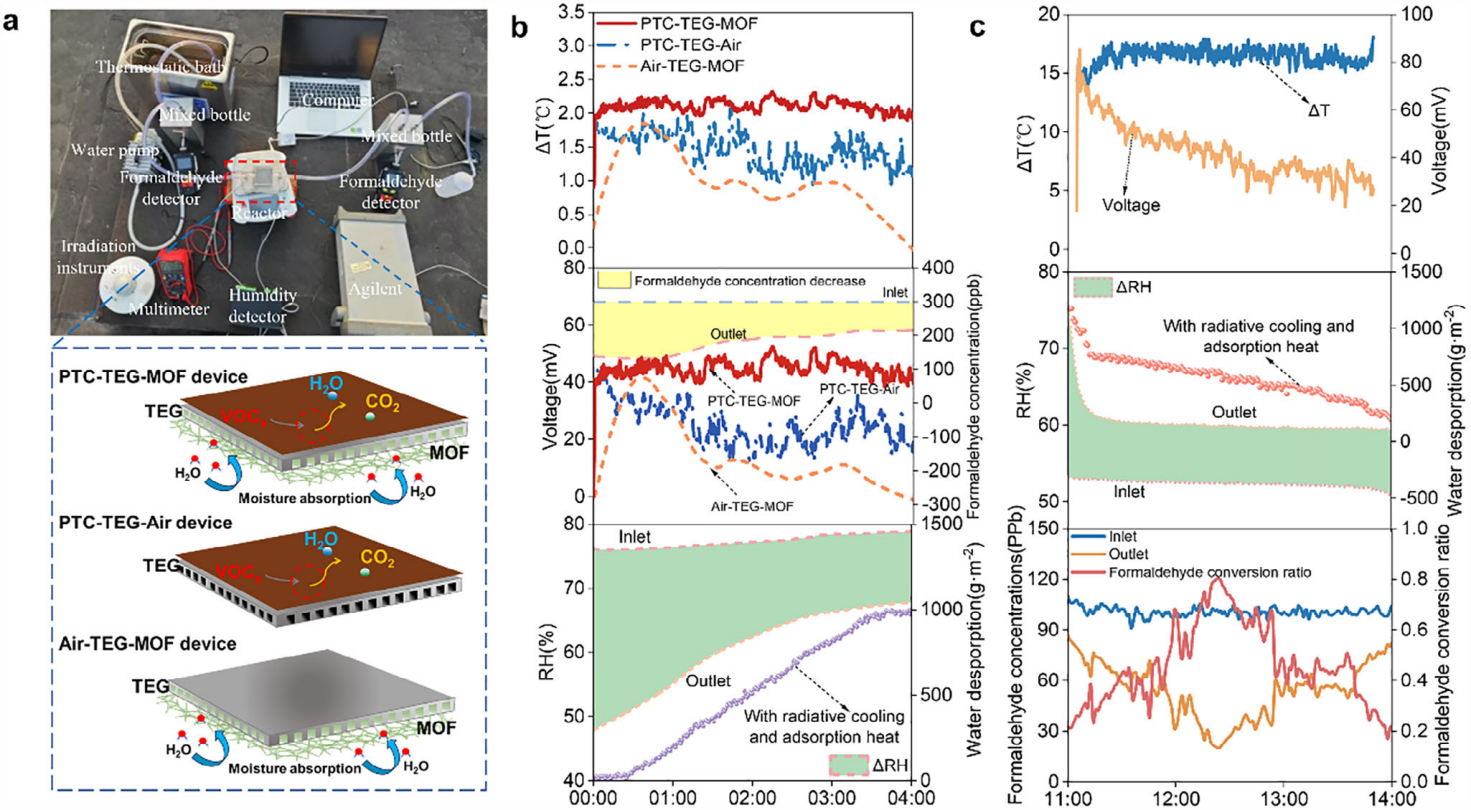
Outdoor demonstration of the hybrid PTC‐TEG‐MOF device for air purification, power, and dehumidification. a) The experimental systems of the PTC‐TEG‐MOF device and other two referred devices. b) The temperature diffidence, voltage, relative humidity, formaldehyde concentration, and water desorption during nighttime. c) The temperature diffidence, voltage, relative humidity, formaldehyde concentration, and water desorption during daytime.

We further conducted rooftop outdoor experiments on the Tiangong Building, Nanjing University of Technology to systematically evaluate the individual and synergistic contributions of the photothermal film and MOF to the system performance (**Figure** **4**a). Three devices were tested in parallel: a hybrid PTC‐TEG‐MOF device, a reference PTC‐TEG‐Air device (without MOF), and a reference Air‐TEG‐MOF device (without PTC). The measurements included temperature difference across the TEG, output voltage, formaldehyde degradation efficiency, and relative humidity regulation.

Under nighttime conditions, the hybrid device achieved a maximum temperature difference (ΔT) of 2.15 °C across the TEG module, higher than that of the PTC‐TEG‐Air (1.5 °C) and Air‐TEG‐MOF (1.2 °C) references. The enhanced ΔT led to a higher open‐circuit voltage (*Voc*), with the hybrid device outputting 53 mV, compared to 44 and 47 mV for the references, respectively (Figure 4b). The corresponding power densities were 80 mW·m^−2^ (PTC‐TEG‐MOF), 57 mW·m^−2^ (PTC‐TEG‐Air), and 63 mW·m^−2^ (Air‐TEG‐MOF), with thermoelectric conversion efficiencies of 0.18%, 0.14%, and 0.15%, respectively (Figures S18 and S19, Supporting Information). These results highlight the synergistic effect of both photothermal heating and MOF adsorption heat on thermoelectric performance.

Meanwhile, the MOF enabled continuous moisture capture from ambient air, with an average uptake of 511.9 g·m^−2^ and a rate of 127.78 g·m^−2^·h^−1^ (Figure 4b). The relative humidity (RH) of outlet air decreased from 77.4% (inlet) to 60.6%, achieving a 16.8% RH drop that falls within the ASHRAE thermal comfort range (40%–65%). Moreover, the released adsorption heat slightly increased the outlet air temperature (Figure S20, Supporting Information), contributing to an estimated 13.06% air‐conditioning energy saving, with a unit‐area energy saving of 7.5 kJ·kg^−1^ (Note S4, Supporting Information). In addition to the thermoelectric and dehumidification functions, the hybrid device also demonstrated effective formaldehyde removal during nighttime operation. Benefiting from the PTC film's low‐temperature catalytic activity, the hybrid device achieved an average formaldehyde conversion rate of 40.6% and a CADR of 48.8 m^3^·h^−1^·m^−2^ (Figure 4b).

During daytime operation, the device showed further enhanced performance (Figure 4c). Under an inlet formaldehyde concentration of 100 ppb, the formaldehyde conversion rate increased to 80% and the CADR reached 170.5 m^3^·h^−1^·m^−2^ (Figure S21, Supporting Information). Simultaneously, solar heating raised the MOF temperature, promoting moisture desorption even in the presence of desorption‐induced cooling (Figure S22, Supporting Information). Under the resulting ΔT across the TEG, the Voc peaked at 90.5 mV, corresponding to a power density of 209 mW·m^−2^ and a thermoelectric efficiency of 0.65% (Figures S23 and S24, Supporting Information).

These results collectively demonstrate that the hybrid PTC‐TEG‐MOF device enables efficient photothermal‐electric conversion, VOC degradation, and passive humidity regulation under both daytime and nighttime conditions.

### 2.4 The Energy‐Saving Impact of PTC‐TEG‐MOF Within a Chinese Operational Context

**Figure 5:**
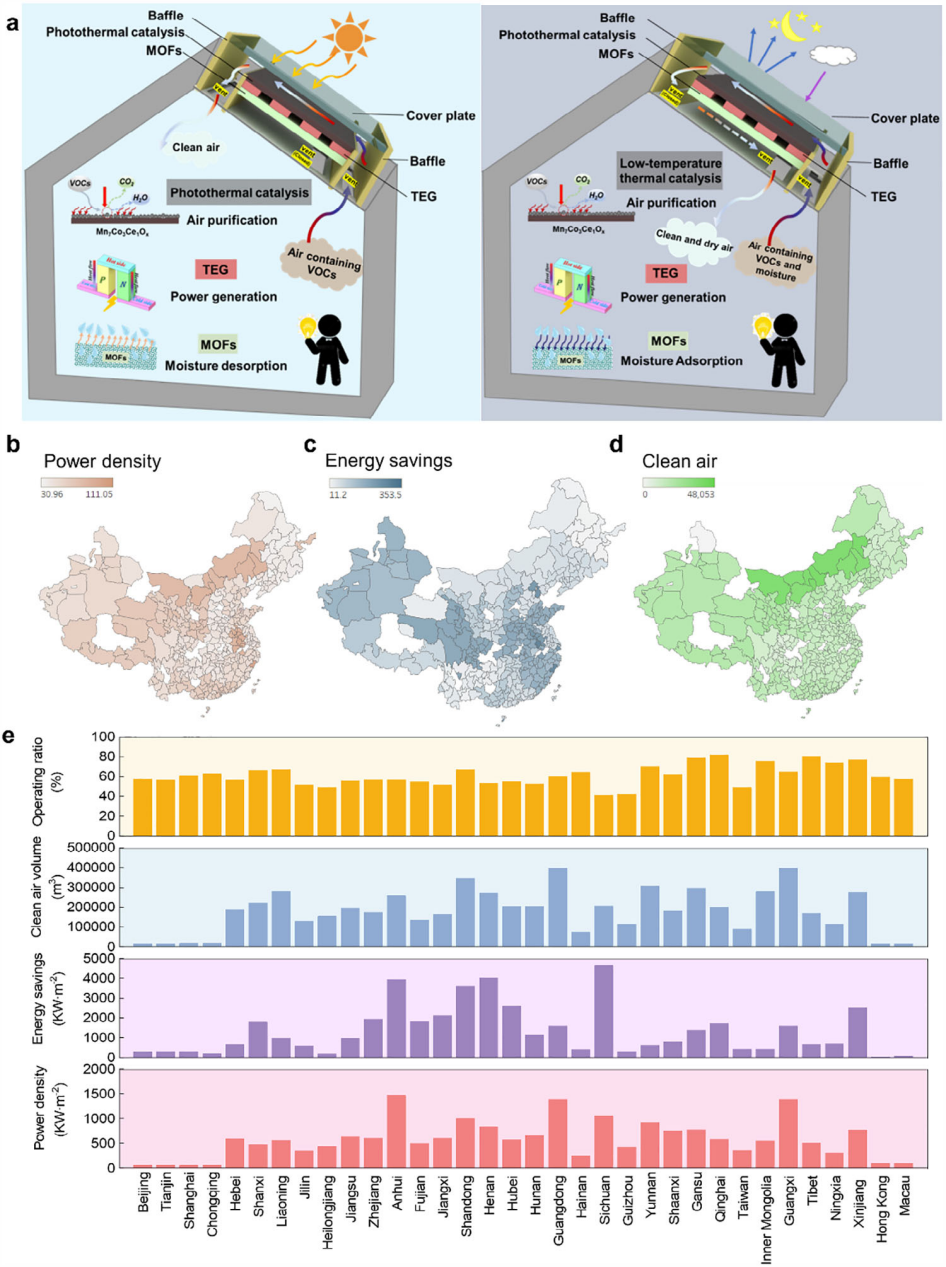
The energy‐saving impact of PTC‐TEG‐MOF within a Chinese operational context. a) The main operating modes of multifunctional roofs: purification‐regeneration mode and purification‐dehumidification mode. b–d) The annual power density, annual air conditioning energy savings, and annual generated clean air in 34 provincial capitals in China. e) The detailed values of the annual operating ratio of purification mode, annual power density, annual air conditioning energy saving, and annual generated clean air in 34 provincial capitals in China.

To evaluate the real‐world applicability and regional adaptability of the multifunctional PTC–TEG–MOF system, we implemented a rooftop installation (5 m × 5 m) on a building and investigated its performance under four typical operation modes:^[^ ^1^ ^]^ purification–dehumidification,^[^ ^2^ ^]^ purification–regeneration,^[^ ^3^ ^]^ non‐purification–desorption, and^[^ ^4^ ^]^ non‐purification–dehumidification (**Figure** **5**a) (Figure S25, Supporting Information). These modes enable dynamic adaptation to varying ambient humidity, temperature, and pollutant concentrations. A comprehensive simulation framework was developed and experimentally validated (Figure S26, Supporting Information), integrating thermal, electrical, dehumidification, and air purification models. Using typical meteorological year (TMY) data from *EnergyPlus*, we analyzed performance in 34 Chinese provincial capitals, assuming a constant indoor temperature of 26 °C, an indoor pollutant level of 100 ppb, and humidity conditions adapted to local climates (Note S1, Supporting Information).

The energy‐saving impact demonstrates the multifunctional roof achieves a generated clean air of 96.3 m^3^·h^−1^·m^−2^ to 1119.3 m^3^·h^−1^·m^−2^, a power generation of 30 kW·m^−2^ to 105.3 kW·m^−2^ and air conditioning energy saving of 11.2 kW·m^−2^ to 353.5 kW·m^−2^ across cities in China (Figure 5b–d). This generated electricity can power household low‐power apparatus such as lighting and sensors. The roof demonstrates good air conditioning energy‐saving performance in high‐humidity and high‐temperature cities such as Wuhan, Zhengzhou, and Jinan, where summer temperature can reach the MOF's regeneration temperature. In contrast, in colder cities like Harbin, Dalian, and Shenyang, summer temperatures rarely achieve this regeneration temperature. The clean air volume is higher in the coastal and northwest regions of the country, as the coastal areas have better air circulation, while the northwest regions have a dry climate and lower pollution levels. The high ambient temperature and sunlight radiation in summer may restrict the purification function to prevent indoor overheating. The annual operating ratio of the purification mode is a key parameter for the energy‐saving performance of multifunctional roofs. The results show that the annual operating ratio of purification mode across cities in China ranges from 41.2% to 82.3%, demonstrating the huge application potential of the device at most times of the year (Figure 5e). In particular, the annual operating ratio is higher in the northwest regions such as Gansu, Ningxia, and Qinghai province. This is mainly due to the higher latitudes and lower solar irradiation in these areas, which allow the roof system to operate efficiently for a longer period, especially in the air purification mode. In general, in the context of Chinese applications, the PTC‐TEG‐MOF device demonstrates significant energy‐saving potential and application prospects. The proposal of the hybrid system presents a new solution for the development of multifunctional building‐integrated systems for air purification, dehumidification, and power generation.

## 3 Discussion

In conclusion, we have made significant contributions in the areas of photothermal composite catalytic material, system innovation, and model development (Figure S27, Supporting Information). First, a novel type‐II heterojunction photothermal catalyst, Mn_7_Co_3_Ce_1_O*_x_*, is developed, which simultaneously offers catalytic activity, photothermal heating, and radiative cooling capabilities.^[^ ^55^ ^]^

This material is developed using innovative synthesis strategies, demonstrating excellent photothermal performance and stability, with a removal efficiency of 90.9%, significantly outperforming traditional photothermal materials, which typically range from 20% to 80% efficiency.^[^ ^56^, ^57^, ^58^, ^59^, ^60^ ^]^ It exhibits a broadband solar absorptance of 94.3% and achieves a rapid surface temperature increase of up to 68 °C under 1000 W·m^−2^ solar irradiation. Notably, it extends the photocatalytic light response range effectively to 1600 nm, delivering an outstanding photothermal catalytic efficiency of 95.4%. Meanwhile, its high solar emissivity of 95.7% within the atmospheric window enables passive radiative cooling under nighttime conditions.

Second, a hybrid system is proposed that combines photothermal catalysis, MOF material moisture absorption, and thermoelectric generation (TEG), which can generate power under all weather conditions and significantly enhances purification, dehumidification, and power generation functions. The system's thermoelectric power density increased by 640.1% due to the synergistic effect, and within 4 h, the air relative humidity was reduced by 16.8%, with a clean air generation rate of 270.8 m^3^·h⁻^1^·m⁻^2^. Compared to existing research,^[^ ^37^, ^38^, ^39^ ^]^ especially when using thermoelectric generation (TEG) alone,^[^ ^61^ ^]^ our system achieves a higher power generation efficiency (0.35%) and a higher power output. Finally, a new non‐steady‐state hybrid model for photothermal catalysis/dehumidification/thermoelectric conversion is introduced, providing theoretical support for multi‐energy conversion systems, with experimental validation confirming the system's efficiency across various operational conditions. These innovations offer new solutions for advancing photothermal catalysis, environmental purification, and energy conversion.

These results demonstrate the hybrid system's great ability to simultaneously generate power and enhance indoor air quality and humidity control. To evaluate real‐world performance at scale, we applied a dynamic, hourly simulation across 34 provincial capital cities in China, based on typical meteorological year (TMY) data. A detailed multiphysics model, calibrated by experimental results, quantified the annual performance of a 5 m × 5 m multifunctional rooftop equipped with thehybrid system. Results show an annual electricity generation potential of 30–105.3 kW·m^−2^, air‐conditioning energy savings of 11.2–353.5 kW·m^−2^, and clean air output ranging from 296.3 to 1119.3 m^3^·h^−1^·m^−2^. To address concerns about indoor overheating during purification‐dominant operation in summer, we introduced a new performance metric—the annual operating ratio of purification mode, which ranges from 41.2% to 82.3% across the country. High ratios observed in regions like Gansu and Qinghai highlight the system's adaptability to diverse climatic conditions.

In this study, we analyzed the policies and practices of various countries regarding the application of solar thermal technology. China has strongly supported the development of solar thermal technology through policies such as the “14th Five‐Year Plan” and the “2030 Carbon Peak Action Plan,” particularly in the fields of building energy efficiency and green buildings. The government promotes the widespread use of solar thermal systems in hot water supply and heating systems through financial subsidies, tax reductions, and other measures. Data from 34 provinces and cities across the country demonstrate the adaptability of solar thermal technology in different regions, proving its vast application potential in China. Additionally, countries such as the United States, Germany, Italy, and India have also driven the development of solar thermal technology through policy support and technological innovation. For example, the U.S. promotes the widespread adoption of solar thermal systems through Federal Investment Tax Credits (ITC) and market incentives, Germany actively supports the technology through green building standards and financial subsidies, and India supports the use of solar thermal systems in rural areas through the National Solar Mission. The practices of these countries demonstrate the promising development prospects of solar thermal technology in various economies and its contribution to the global clean energy transition.^[^ ^62^ ^]^ (Note S8, Supporting Information).

Overall, this work presents a novel strategy for integrated building‐environmental management, offering a low‐energy, low‐carbon, and multifunctional solution for simultaneous power generation, air purification, and humidity control. The proposed hybrid design paves the way for sustainable, climate‐adaptive building systems, and demonstrates high application potential across varied geographic and climatic contexts. Future research directions could focus on further optimizing the performance of photothermal catalytic materials to enhance their stability and efficiency. Additionally, exploring the integration and application of the system in different building scales and climatic conditions will ensure its versatility and long‐term reliability. Moreover, integrating IoT technologies to develop smart control systems for optimizing energy management could enable more efficient air purification, power generation, and humidity control. Finally, promoting interdisciplinary collaboration to drive the fusion of architecture, energy, and environmental technologies will facilitate the innovative development of sustainable building systems.

## 4 Experimental Section

### Preparation of Photothermal Catalyst

The preparation method of Mn_7_Co_3_Ce_1_O_x_: Mn(CH_3_COO)_2_·4H_2_O, Ce(NO_3_)_3_·6H_2_O, and Co(NO_3_)_2_·6H_2_O with a total amount of 0.0097 mol were dissolved in 100 mL deionized water at a ratio of 9: 3: 1 and stirred for 30 min. At the same time, 0.001 mol of Na_2_CO_3_ was added to 10 mL of deionized water, so that it was fully dissolved and slowly added to the mixed solution, and continued to stir for 2 h. After stirring, the precipitate was centrifuged in a centrifuge and dried at 110 °C for 12 h. After drying and grinding, it was placed in a tube furnace and calcined at 400 °C for 5 h (Figure S28, Supporting Information).

### Characterizations of Mn_7_Co_3_Ce_1_O_x_ Composite

The X‐ray diffraction (XRD) patterns were obtained by a Rigaku Ultima IV X‐ray diffractometer Rigaku, with Cu Kα radiation at 50 kV and 25 mA, scanning at a rate of 5° min^−1^. Scanning Electron Microscopy (SEM) was performed using a Hitachi Regulus8100, Sigma 300 (Japan). Transmission Electron Microscope (TEM) was tested by FEI Talos F200x. The absorptivity/emissivity in the range of 0–25 µm was measured using the Nicolet iS50 spectrometer based on the integrating sphere method. The characteristic signal of superoxide anion radical was obtained by Bruker EMXplus‐6/1 under dark and light conditions with a full‐spectra Xe lamp. The photocurrent and resistance were analyzed through photoelectric testing. A 10 mg sample was dispersed in 1 mL of ultrapure water/ethanol solution with 50 µL of Nafion, sonicated for 30 min, drop‐cast onto an ITO substrate, and dried at room temperature prior to measurement. The UV–vis–NIR spectrophotometer used for measuring spectrum absorptivity and band gap was a Shimadzu UV‐3600i Plus (Japan).

### Preparation of MOF Composite

Hydrothermal synthesis of MIL‐101 (Cr): Cl_3_CrH_12_O_6_ and C_6_H_4_‐1,4‐(CO_2_H)_2_ with a total amount of 0.0141 mol were dissolved in 50.4 mL deionized water in a ratio of 0.986: 1 to form a mixed liquid, which was magnetically stirred at room temperature for 30 min. The mixed liquid was loaded into a polytetrafluoroethylene autoclave (100 mL) and hydrothermally heated at 220 °C for 24 h. The obtained solution was cooled to room temperature and then centrifuged for 10 min to collect the product. Finally, the MIL‐101 (Cr) powder was obtained by washing with DMF and ethanol, respectively (Figure S29, Supporting Information).

### Performance of Hybrid PTC‐TEG‐MOF Device

Indoor and outdoor experiments were carried out to evaluate the performance of a hybrid PTC‐TEG‐MOF device. For the indoor experiments, all proof‐of‐concept experiments for the hybrid device were conducted at controlled temperatures and relative humidity, with fluctuations in temperature and relative humidity kept below 1 °C and 3%, as measured by a temperature sensor (K‐Type Thermocouple) and humidity sensor (KS‐SH51AP). Indoor simulated nighttime experiments used water‐cooled panels to simulate radiative cooling to accelerate the hygroscopicity of the hybrid device at 28 °C and 80% RH. Indoor simulated daytime experiments were conducted at light intensities of 500, 800, and 1000W·m^−2^ using a sunlight simulator for moisture desorption with uniformity of 10%, a temporal instability of 10% and a spectral match classification of AAA. For the outdoor experiments, the hybrid device was placed on the roof of the Tiangong Building at Nanjing Tech University, and a transparent polyethylene membrane was used to suppress convective heat loss from the hybrid device. At night, moisture adsorption occurred from 00:00 AM to 04:00 AM (UTC+8), and radiative cooling enhanced moisture adsorption and power generation. During the day, moisture desorption and formaldehyde purification occur by driving the hybrid device between 11:00 AM and 14:00 PM (UTC+8), and the formaldehyde concentration was detected by the formaldehyde detector (HCX400‐CH_2_O). The solar flux was measured in real‐time by a solar intensity meter. In contrast, a reference device without MIL‐101 (Cr) adsorbent was prepared and tested to evaluate the effect of moisture adsorption/desorption on power generation performance. Changes in temperature and relative humidity were measured in real‐time by installing temperature and relative humidity sensors inside the hybrid device (Note S2 and Figure S30, Supporting Information).

### Statistical Analysis

In order to obtain the error and accuracy of the experimental results, the uncertainty of the experiment results was calculated from two aspects of absolute error and relative error. The expression of uncertainty is expressed as follows:

(1) $$\Delta y=\sqrt{{\left(\frac{\partial f}{\partial x_{1}}\right)}^{2}\Delta {x_{1}}^{2}+{\left(\frac{\partial f}{\partial x_{2}}\right)}^{2}\Delta {x_{2}}^{2}+\cdots +{\left(\frac{\partial f}{\partial x_{n}}\right)}^{2}\Delta {x_{n}}^{2}}$$

(2) $$\begin{array}{c} \frac{\Delta y}{y}=\sqrt{{\left(\frac{\partial f}{\partial x_{1}}\right)}^{2}{\left(\frac{\Delta x_{1}}{y}\right)}^{2}+{\left(\frac{\partial f}{\partial x_{2}}\right)}^{2}{\left(\frac{\Delta x_{2}}{y}\right)}^{2}+\cdots +{\left(\frac{\partial f}{\partial x_{n}}\right)}^{2}{\left(\frac{\Delta x_{n}}{y}\right)}^{2}} \end{array}$$

where *x* is the independent variables, *f* is the function associated with variables. By calculation, the experimental uncertainty of each result during the experiment are between 4.2% and 6.5%, the average uncertainty is 5.3%, which indicates that the experimental results are reliable (Figure S31, Supporting Information).

## Conflict of Interest

The authors declare no conflict of interest.

## Supporting information



Supporting Information

## Data Availability

The data that support the findings of this study are available on request from the corresponding author. The data are not publicly available due to privacy or ethical restrictions.
