# Supplementary material for: A Multifunctional Photothermal Catalyst Enabling Full‐Day Sustainable Power and Indoor Air Quality Control
Source: Adv Sci (Weinh). 2025 Jun 20;12(34):e05059. doi: 10.1002/advs.202505059 (PMC12442673; doi:10.1002/advs.202505059)
Supplement: Supplementary file 1 — Supporting Information [file ADVS-12-e05059-s001.docx]

**Supplemental material for**

**A Multifunctional Photothermal Catalyst Enabling Full-day Sustainable Power and Indoor Air Quality Control**

Niansi Li ^1, #^; Wei Wei ^1, #^; Yulin Li ^1, #^; Feiyang Xu ^1, #^; Guoyu Zhang ^1^; Jie Ji ^2^; Xudong Zhao ^3^; Junwei Liu ^4^; Bendong Yu ^1, *^; Qiliang Wang ^5, *^

1. College of Urban Construction, Nanjing Tech University, Nanjing 210009, Jiangsu, China

2. Department of Thermal Science and Energy Engineering, University of Science and Technology of China, Hefei 230026, China

3. Centre for Sustainable Energy Technologies, University of Hull, Hull HU6 7RX, United Kingdom

4. Department of Building Environment and Energy Engineering, The Hong Kong Polytechnic University, Kowloon, Hong Kong

5. Department of Architecture and Built Environment, University of Nottingham, University Park, Nottingham, NG7 2RD, United Kingdom

^#^ These authors contribute equally to this work

^*^ Correspondence: [yubendonglns@163.com](mailto:yubendonglns@163.com); [bendongyu@njtech.edu.cn](mailto:bendongyu@njtech.edu.cn) (B. Y.); [qiliang.wang@ustc.edu](mailto:qiliang.wang@ustc.edu) (Q. W.)

**Table of Contents Supplementary Notes**

**Supplementary Note 1:** Theoretical analysis of the energy balance of hybrid PTC-TEG-MOF for formaldehyde purification and electricity co-generation

**Supplementary Note 2:** Performance of PTC-TEG-PCM hybrid device

**Supplementary Note 3:** Model verification

**Supplementary Note 4.** Energy consumption analysis

**Supplementary Note 5.** Evaluation parameters

**Supplementary Note 6.** Seasonal analysis

**Supplementary Note 7.** Hydroscopic material

**Supplementary Note 8.** Method

**Supplementary Note 9.** Sensitivity calculation analysis

**Supplementary Note 1.**

**Theoretical analysis of the energy balance of hybrid PTC-TEG-MOF for formaldehyde purification and electricity co-generation**

Compared with the photovoltaic system alone, the PTC-TEG-PCM hybrid system was more complex in structure. In order to simplify, the following assumptions were used in the numerical simulation(*1*)**：**

**(1)** The simulation was based on one-dimensional and steady-state heat transfer processes. On the surface of the same height, the heat flux and temperature values were uniform(*2*).

**(2)** The energy loss only occurred on the top surface of the PV and the bottom surface of the PCM device, while the heat loss from the system through the module side to the environment was ignored.

**(3)** The physical parameters of the material in the system, including the TE characteristics of n-type and p-type semiconductors in TEG, were constant and temperature-independent.

**(4)** The thermal contact resistance between adjacent layers in the system was an appropriate constant, which was determined by the thickness of the air layer.

**(5)** The thermal radiation loss between the phase change device and the ground was zero, and the temperature difference was small(*3*).

The hourly weather data for 34 cities were provided by typical meteorological year (TMY) data from ***EnergyPlus*** software. The indoor temperature is set at 26°C, with the indoor relative humidity adjusted based on the average outdoor relative humidity. The indoor pollutant concentration is 100 ppb.

In order to analyze, predict and optimize the performance of the system, we set up the thermal model and the purification model of the PTC-TEG-PCM system, and verified the accuracy of the established system model by comparing the all-day dynamic experimental data with calculation results of the model. The thermoelectric transfer model consisted of five parts, which are glass cover plate, air flow channel, thermal catalytic coating, thermoelectric plate, and phase change material layer, respectively. The mass transfer model mainly included the degradation of gaseous formaldehyde. The subscripts of *glass*, *ar*, *ptc*, *teg* and *mof* represents glass cover plate, air flow channel, photothermal catalytic layer, thermoelectric plate, and phase change material layer, respectively. The mass transfer model mainly studies the problem of formaldehyde purification. The establishment of the model is based on two basic assumptions: (1) All physical parameters are assumed to be constant; (2) Considering the low concentration of formaldehyde in the actual situation, the reaction heat generated by formaldehyde in the catalytic reaction process is ignored. Some important assumptions set in the model could be referred to previous research work (*4*).

The equations relating to glass are as follows:

$T_{sky}=0.0552T_{outdoor}^{1.5}$ (1)

$\begin{matrix} m_{glass}c_{glass}\frac{dT_{glass}}{dt}=GA_{glass}\alpha_{glass}+h_{outdoor,glass}A_{glass}\left(T_{outdoor}-T_{glass}\right) \\ +h_{sky,glass}A_{glass}\left(T_{sky}-T_{glass}\right)+h_{pc,glass}A_{glass}\left(T_{pc}-T_{glass}\right) \\ +h_{ar,glass}A_{glass}\left(T_{ar}-T_{glass}\right) \end{matrix}$ (2)

$h_{sky,glass}=\sigma\varepsilon_{glass}(T_{sky}+T_{glass})(T_{sky}^{2}+T_{glass}^{2})$ (3)

$h_{outdoor,glass}=6.5+3.3v_{outdoor}$ (4)

$\left\{\begin{matrix} Nu=0.68+\frac{0.670Ra^{\frac{1}{4}}}{[1+(0.492/\Pr)^{\frac{9}{16}}]^{\frac{4}{9}}}\begin{matrix} & & \end{matrix}\begin{matrix} Ra\leq10^{9} & \end{matrix} \\ Nu=\left(0.825+\frac{0.387Ra^{\frac{1}{4}}}{[1+(0.492/\Pr)^{\frac{8}{27}}]^{\frac{4}{9}}}\right)^{2}\begin{matrix} & Ra>10^{9} & \end{matrix} \end{matrix}\right.$ (5)

$h_{ar,glass}=\frac{Nu\cdot\lambda_{glass}}{L_{c}}$ (6)

$h_{pc,glass}=\sigma\varepsilon_{glass}(T_{pc}+T_{glass})(T_{pc}^{2}+T_{glass}^{2})$ (7)

where *m_glass_*, kg, is the mass of the glass cover plate node. *c_glass_*, J/(kg·K), is the specific heat capacity. A_glass_, m^2^, is the area, α_glass_ is the absorption rate. *T_sky_*, *T_glass_*, *T_outdoor_*, *T_ar_* and *T_ptc_*, K, are the temperatures of the sky, glass, outdoor environment, air in the airflow channel and the photothermal catalysis layer, respectively. *h_sky, glass_*, *h_outdoor, glass_*, *h_ar, glass_* and *h_PTC, glass_*, m/s respectively represent the radiative heat transfer coefficient or convective heat transfer coefficient between the glass and the sky, the outdoor environment, the air in the channel and the catalytic layer. *G*, W/m^2^, is the solar radiation intensity received by the system. *L_c_*, m, the height of the air passage.

The heat balance equation of the air in the air channel is as follows:

$m_{ar}c_{ar}\frac{\partial T_{ar}}{\partial t}=h_{ar,glass}A_{ar}\left(T_{glass}-T_{ar}\right)+h_{p\text{t}c,ar}A_{ar}\left(T_{p\text{t}c}-T_{ar}\right)-m_{ar}c_{ar}v_{ar}\frac{\partial T_{ar}}{\partial y}$ (8)

$v_{ar}=\sqrt{\frac{g\beta(T_{ar\_out}-T_{ar\_in})L_{c}}{f(L_{c}/d_{c})}}$ (9)

where *m_ar_*, *c_ar_* and *A_ar_*, are represent the mass, specific heat capacity and area of the air, the unit are kg, J/(kg·K) and m^2^, respectively. *v_ar_*, m/s, represents air flow velocity, and its calculation under natural convection conditions is based on the pressure balance, the formula is shown in Equation (9). Where *g,* m/s^2^, is the acceleration of gravity. *β, K*^-1^*,* is the air expansion coefficient. *f_in_*, *f_out_* and *f* are the inlet, outlet local resistance coefficients and the friction loss coefficient.

The mass balance equation of the air in the air channel is as follows:

$\frac{\partial C_{m}}{\partial t}+v_{ar}\frac{\partial C_{m}}{\partial t}+h_{m}(C_{s}-C_{m})=0$ (10)

$C_{s}=\frac{h_{m}C_{m}}{(k_{app}+h_{m})}=\frac{r}{k_{app}}$ (11)

$h_{m}=Sh\cdot1.86\times10^{-5}/L_{c}$ (12)

$Sh=Nu\cdot Le^{1/3}$ (13)

where *C_m_* and *C_s_*, ppb, are air mainstream formaldehyde concentration and formaldehyde concentration on thermal catalyst surface, respectively. *C_s_* can be calculated by the Equation (11). *h_m_*, m/s, is the mass transfer coefficient of gaseous formaldehyde on catalyst surface, which can be calculated *Sh*, the expression as Equation (12). The *Sh* represents the ratio of convective mass transfer to diffusion mass transfer, which can be calculated by *Nu* and *Le*, the expression as Equation (13). *k_app_*, m/s, is the apparent reaction coefficient, which can be calculated by *C_s_* and *r*. *r*, ppb·m/s, is the reaction rate, which can be obtained by kinetic model. The kinetic model can be seen in Ref. (*5*).

The equation relating to the photothermal catalytic layer is as follows:

$\begin{matrix} m_{\text{ptc}}c_{\text{ptc}}\frac{\partial T_{\text{ptc}}}{\partial t}=\lambda_{\text{ptc}}A_{\text{ptc}}\frac{\partial^{2}T_{\text{ptc}}}{\partial y^{2}}+GA_{\text{ptc}}\tau_{g\text{lass}}\alpha_{\text{ptc}}+h_{\text{ptc}-g\text{las}s}A_{\text{ptc}}\left(T_{g\text{las}s}-T_{\text{ptc}}\right) \\ +h_{\text{ptc}-ar}A_{\text{ptc}}\left(T_{ar}-T_{\text{ptc}}\right)+\frac{T_{\text{teg}}-T_{\text{pyc}}}{R_{\text{teg}-\text{ptc}}} \end{matrix}$ (14)

where ***m_ptc_***, ***c_ptc_***, ***A_ptc_***, and ***α_ptc_***, are represent the mass, specific heat capacity, area, absorptivity of the photothermal catalytic layer, the unit are kg, J/(kg·K), m^2^, respectively. ***τ_glas_****_s_* is the transmittance of glazing cover. ***R_teg-ptc_***, m^2^·K/W, is the thermal conductivity resistance of the TEG panel.

Atmospheric emissivity was relatively complex due to the differences in atmospheric composition, and weather and climatic conditions. Here, we used Equation (27) to derive the atmospheric emissivity(*6-8*).

$\varepsilon_{r}=0.711+0.56\left(\frac{T_{dew}}{100}\right)+0.73\left(\frac{T_{dew}}{100}\right)^{2}$ (15)

where ***T_dew_*** was the local dew point temperature.

***E_b_ (λ, T)*** could be calculated as follows(*8*):

$E_{b}\left(\lambda,T\right)=\frac{2hc^{2}}{\lambda^{5}}\frac{1}{e^{hc/\lambda k_{B}T}-1}$ (16)

where ***ε (λ, θr)*** was the emissivity of the radiative cooling paint dependent on the solar incidence angle ***θs***; ***E_AM1.5_*** was solar radiation intensity with normal solar spectrum; ***h*** was Planck's constant of 6.63×10^−34^ J s; ***c*** was the speed of light in vacuum; and ***k_B_*** was Boltzmann's constant of 5.67×10^−8^ W/m^2^ K^4^.

The energy balance function for the TEG panel is given as(*9*):

$\begin{matrix} \text{ρ}_{\text{teg}}\text{δ}_{\text{teg}}\text{c}_{\text{teg}}\frac{\partial\text{T}_{\text{teg}}}{\partial\text{t}}=\text{G}\tau_{g\text{lass}}\tau_{\text{ptc}}\alpha_{\text{teg}}-Q_{\text{teg}}+\text{h}_{\text{ptc-teg}}\left(\text{T}_{\text{ptc}}-\text{T}_{\text{teg}}\right)+\text{h}_{\text{r}\_\text{tegmof}}\left(\text{T}_{\text{mof}}-\text{T}_{\text{teg}}\right) \\ \text{ }+\frac{\text{k}_{\text{teg}}}{\text{δ}_{\text{teg}}}\left(\text{T}_{\text{ptc}}-\text{T}_{\text{teg}}\right)\text{+}\frac{\text{k}_{\text{teg}}}{\text{δ}_{\text{teg}}}\left(\text{T}_{\text{mof}}\text{-T}_{\text{teg}}\right) \end{matrix}$ (17)

where ***ρ_teg_*** is density of TEG panel (kg·m^-3^); ***δ_teg_*** is thickness of the TEG panel (m); ***c_teg_*** is the capacity of the TEG panel (J·K^-1^·m^-2^)(*10*); ***Q_teg_*** is the output power of TEG panel; ***h_r_tegmof_*** is the radiant heat transfer coefficient between the TEG panel and MOF (W·m^-^²·K^-1^) and could be calculated via the emissivity of the object, as showed in Eq.(18), where ***ε_mof_*** is emissivity of the MOF(*11*) ; ***k_teg_*** is thermal conductivity of TEG panel (W·m^-^²·K^-1^).

$h_{\text{ptc-}teg}=\frac{\sigma\left(T_{\text{teg}}+T_{\text{ptc}}\right)\left(T_{\text{teg}}^{2}+T_{\text{ptc}}^{2}\right)}{1/\varepsilon_{\text{teg}}+1/\varepsilon_{\text{ptc}}}$ (18)

$h_{r\_\text{tegmof}}=\frac{\sigma\left(T_{\text{teg}}+T_{\text{mof}}\right)\left(T_{\text{teg}}^{2}+T_{\text{mof}}^{2}\right)}{1/\varepsilon_{\text{teg}}+1/\varepsilon_{\text{mof}}}$ (19)

The heat and mass transfer equation of wet air channel can be calculated according to the following formulas:

$\frac{1}{u_{m}}\frac{\partial T_{a}}{\partial t}+\frac{\partial T_{a}}{\partial y}=\frac{4h}{d_{e}u_{m}\rho_{a}c_{a}}\left(T_{mof}-T_{a}\right)$ (20)

$\frac{1}{u_{m}}\frac{\partial\left(d_{a}\right)}{\partial t}+\frac{\partial\left(d_{a}\right)}{\partial y}=\frac{4h_{m}}{d_{e}u_{m}}\left(d_{mof}-d_{a}\right)$ (21)

where ***ρ_a_*** was density of moist air (kg·m^-3^); ***c_a_*** was the capacity of moist air (J·K^-1^·m^-2^); u_m_ was the average velocity of the airflow (m/s); ***d_a_*** was moisture content of airflow(g/kg); ***d_mof_*** was moisture content of MOF(g/kg); ***d_e_*** was flux equivalent diameter(m); ***y*** was axial coordinates along the flow channel direction (m); ***h*** was convective heat transfer coefficient between moist air and MOF; ***h_m_*** was convective mass transfer coefficient between moist air and MOF; Le^2/3^ was the Lewis number.

$h_{m}=\frac{h}{\rho_{a}c_{a}Le^{2/3}}$ (22)

The following equation is the heat and mass transfer equation of MOF:

$\rho_{d}c_{tot}\frac{\partial T_{mof}}{\partial t}=k_{d}\left(\frac{\partial T_{mof}}{\partial x}+\frac{\partial T_{mof}}{\partial y}\right)+q_{st}\rho_{d}\frac{\delta w}{\delta t}$ (23)

where ***ρ_d_*** was density of dry absorbent(kg/m^3^) ; ***k_d_*** was thermal conductivity of dry absorbent (W/(m·K)); ***w*** was moisture content of hygroscopic agent(kg/kg); ***q_st_*** was adsorption heat(kJ/kg); ***c_tot_*** was the total heat capacity of MOF materials(kJ/kg).

$c_{tot}=c_{pd}+wc_{pw}$ (24)

$w=\frac{f_{d}W_{max}}{1-C+C/RH}$ (25)

where ***W_max_*** was maximum adsorption capacity(kg/kg); ***C*** was a constant, which determined the shape of the adsorption isotherm. RH was relative humidity; ***f_d_*** was mass ratio of effective adsorbent.

For room air, the four-node method is adopted in the modeling of room air, and the heat balance equation for the room air can be expressed as follows [45]:

$\rho c\frac{dT_{r}}{dt}W_{r}L_{r}H_{r}=2\rho cAV_{a}\left(T_{out}-T_{in}\right)-hc_{c}\left(T_{out}-T_{c}\right)W_{r}L_{r}+\sum_{i=1}^{n}hc_{i}\left(T_{i}-T_{r}\right)A_{i}$ (26)

where $W_{r}$ is width of the room, m; $L_{r}$ is length of the room, m; $H_{r}$ is height of the room, m; $hc_{c}$ is the convective heat transfer coefﬁcient between the outlet air and the ceiling, $W/m^{2}\cdot K$; $hc_{i}$ is the convective heat transfer coefficient of each inner surface, $W/m^{2}\cdot K$; and $i$ represents the interior surface of each room in the room.

$hc_{c}$ can be calculated by the air exchange rate ACH, which is expressed as [46],

$hc_{c}=-0.166+0.484ACH^{0.8}$ (27)

After the heat transfer model was established, the energy conservation equations of each part were discretized respectively. The outdoor meteorological parameters measured during the experiment were taken as the boundary conditions of the model. The parameters included solar radiation, outdoor air temperature, and outdoor velocity. Finally, the meteorological parameters were compiled into MATLAB code file and imported into the software for calculation. The initial values of the temperature parameters of each part were the temperatures measured before the start of the experiment. According to the calculation process, the temperatures of each part were calculated in turn. All the physical parameters used in the calculation were shown in supplementary table 1.

In addition, in order to analyze the accuracy of the model established, the root mean square deviation (RSMD) was used to calculate the errors of the model and the experimental results. The RSMD expression is as follows:

$RSMD=\sqrt{\frac{\sum[(X_{mol,i}-X_{\exp,i})/X_{\exp,i}]^{2}}{n}}$ (28)

where *X_mod,i_* and *X_exp,i_* represent the model calculation results and experimental results of a certain parameter respectively, and *n* represents the total number of parameters.

**Supplementary Note 2.**

**Performance of PTC-TEG-PCM hybrid device**

**Steady state experiment (hot and cold)**

The steady-state experiments on electricity, heat, and radiative cooling were conducted under constant illumination and cold-end conditions. These experiments were carried out in the laboratory at Nanjing Tech University. First, a xenon lamp was used as a simulated light source to replicate the temperature changes of the PTC composite panel, thermoelectric generator, and MOF material under different irradiation intensities (500W/m², 800W/m², and 1000W/m²). The single-pass rate of the hybrid system, the electrical output of the TEG panel, and the water precipitated by the MOF material were also measured. Additionally, to simulate the radiative cooling effect of the PTC material at night, the constant-temperature cold water was used to simulate the radiation effect of the atmospheric window band in order to achieve auxiliary cooling. The structure of the hybrid system varied between day and night to optimize performance. During daytime, the components were arranged from top to bottom as follows: a xenon lamp, a PTC composite panel(60mm×60mm), a TEG panel(50mm×50mm), and MOF materials(50mm×50mm). At night, the system was configured with an auxiliary cooling module (iron cooling sheet) on top, followed by a photothermal composite catalytic layer, a TEG panel, and MOF materials. To maintain a stable temperature of 20°C at night, a water pump circulated cold water from a thermostatically controlled low-temperature tank, ensuring effective cooling.

**Unsteady state experiment (hot and cold)**

The outdoor experiment was conducted to evaluate the electrical, thermal, and radiative cooling performance under unsteady-state conditions. The PTC-TEG-MOF hybrid system is structured from top to bottom as follows: a radiative cooling PDMS membrane, an air channel made of PDSM membrane and acrylic glass (60mm×60mm×15mm), an aluminum plate coated with photothermal catalytic material (60mm×60mm×1mm), a thermoelectric generation module (50mm×50mm), and a layer containing MOF materials (50mm×50mm×20mm). Thermal silicone was used to connect the components to enhance thermal conductivity. To reduce heat loss and ensure more accurate performance measurements, the side walls of the experimental setup were insulated.

The automatic weather station was used to monitor the ambient temperature and wind speed, and the solar irradiation intensity was recorded by the irradiator(*12*). A pyranometer was employed to record the solar radiation intensity. The temperatures of the various components of the PTC-TEG-MOF system were measured using K-type thermocouples connected to a data acquisition system, while the output voltage of the thermoelectric module was directly recorded using a multimeter. Humidity levels were measured with a humidity tester. The data acquisition system recorded the measurements at 10-second intervals. Both the automatic weather station and temperature data were recorded at intervals of 10 seconds, while humidity and voltage were recorded intervals of 60 seconds. The model and accuracy of the experimental instruments were shown in Table 1.

**Supplementary Note 3.**

**Model verification**

The results of the experiment and calculation were basically consistent, which demonstrated that the correction method was reliable and feasible. The effectiveness of mathematical models in analyzing existing systems depended largely on their ability to accurately replicate observed field conditions(*13*). Model calibration of thermal conditions was the process of selecting model parameters to reduce the deviation between the model and the observed parameters(*14*). This verification was designed to confirm whether the results of the system in long-term operation were consistent with expectations, and whether they met the assumptions and parameter settings of the model(*15*). The experimental and simulation results of each part, and clearly showed the good consistency between the simulation and experimental results, which indicated that the established model was accurate.

**Supplementary Note 4.**

**Energy consumption analysis**

The energy demand of an air conditioning system is obtained using the thermodynamic properties of air, a method that provides a more intuitive picture of the energy demand of a complex air conditioning system. The ambient conditions of the applied unit are the average temperature after PTC-TEG-MOF treatment 30.7℃ and the average humidity RH 64.1%. The ambient conditions of the non-applied unit are the average temperature of the outdoor air 28.5℃ and the average humidity RH 78.5%. The energy demand can be calculated by the following equation:

 (29)

where ***Q*** is Total energy demand, kW; ***m*** is mass flow rate of air, 0.0010kg/s; ***h*** is enthalpy difference between air entering and leaving the air-conditioning system.

The total enthalpy of air can be expressed as the sum of the enthalpy of dry air and the enthalpy of water vapor.

$h=h_{air}+W\times h_{vapor}$ (30)

where ***h*** is the total enthalpy of wet air, kJ/kg; ***h_air_*** and ***h_vapor_*** are enthalpy of dry air and the specific enthalpy of water vapor; W is absolute humidity.

$W=\frac{0.622\times RH\times P_{sat}\left(T\right)}{P-RH\times P_{sat}\left(T\right)}$ (31)

where ***P_sat_***(T) is the saturated vapor pressure of water at temperature (T).

**Supplementary Note 5.**

**Evaluation parameters**

In order to evaluate the energy-saving potential of the system, we evaluated the thermal performance and formaldehyde degradation performance of the system. For the thermal performance of system, only the heat gain of the air in the air channel was considered to calculate the thermal efficiency of the system. The instantaneous and average thermal efficiency of the system can be expressed as follows:

$\eta_{t}=\frac{m_{ar}c_{ar}(T_{ar\_out}-T_{ar\_in})}{A_{tc}G}$ (32)

$\overline{\eta_{t}}=\frac{\int m_{ar}c_{ar}(T_{ar\_out}-T_{ar\_in})dt}{\int A_{tc}Gdt}$ (33)

where *m_ar_*, kg/s, is the air mass flow rate in the airflow channel. *c_ar_*, j/(kg·K), is the specific heat capacity of air. *T_ar_in_* and *T_ar_out_*, °C, are inlet and outlet temperatures of the airflow channel, respectively. *A_tc_*, m^2^, is the area receiving solar radiation. *G*, W/m^2^, is the solar radiation intensity.

For the formaldehyde degradation performance of the system, we adopted the degradation efficiency of formaldehyde single-through the catalyst surface *η_f_* and the clean air delivery (*CADR*) as the two main evaluation indexes, and the expressions are as follows:

$\eta_{f}=\frac{C_{in}-C_{out}}{C_{in}}$ (34)

$CADR=Q_{ar}\cdot\eta_{f}=v_{ar}\cdot A_{tc}\cdot\eta_{f}$ (35)

$V_{c}=\int CADRdt$ (36)

where *C_in_* and *C_out_*, ppb, are the inlet and outlet formaldehyde concentration in the air channel, respectively. *V_c_*, m^3^, is the volume of clean air produced by the system, which can be obtained by CADR integration.

***η_teg_*** is the conversion efficiency of TEG panel, is given as(*16-18*):

$\eta_{teg}=\left(1-T_{cold}/T_{hot}\right)\times\frac{\sqrt{1+ZT_{teg}}-1}{\sqrt{1+ZT_{teg}}+T_{cold}/T_{hot}}$ (37)

where ***T_cold_*** and ***T_hot_*** are the temperatures at the cold and hot sides of the TEG panel respectively; ***T_teg_*** is the average temperature of the TEG panel and ***Z*** is taken as a common value achieved by the commercial TEG products, Z = 0.004 K^-1^(*19*).

At night, ***V_teg_*** could be calculated by the following formula(*20*):

$V_{teg}=\Delta TS\eta_{teg}$ (38)

Where ***ΔT*** is the temperature difference between the hot side and the cold side; ***S*** is the Seebeck coefficient(*21, 22*), which is 10V/K according to the selected material.

Areal power density (***P_density_***) during night was calculated by the equation(*23*): ←

$P_{\text{density}}=V_{\text{oc}}^{2}/(4\times R_{\text{module}}\times A_{\text{module}})$ (39)

where ***Voc***, ***R_module_***, and ***A_module_*** were the measured open-circuit voltage, resistance, and the area of TEG module.

The core principle of radiative cooling is that an object dissipates heat to the external environment by emitting infrared radiation. This process typically involves the material's radiative properties and the ambient temperature. The Stefan-Boltzmann law is commonly used to calculate radiative cooling, and its formula is as follows:

$Q_{\text{rad}}=\varepsilon_{\text{ptc}}\sigma A(T_{\text{ptc}}^{4}-T_{\text{sky}}^{4})$ (40)

Where ***ε_ptc_*** is the emissivity of material in the atmospheric window; ***σ*** is the Stefan-Boltzmann constant, 5.67×10^-8^ W/m^2^⋅K^-4^; ***A*** is the surface area of the material, m^2^.

Water adsorption capacities of the MIL-101(Cr) sorbent and its composite were studied by a gravimetric method using a precision electronic balance with a resolution of 0.01g Prior to experiments, the MIL-101(Cr) sorbent and its composite samples were dried at 105°C for 12h to determine the dry MIL-101(Cr) coating mass, ***m_coating_*** (g). The mass fraction of the pure MIL-101(Cr) sorbent in the coating can be calculated based on the slurry recipe. The coated sample was then cooled down and placed on the balance at a predetermined environment condition. The mass change ***∆m*** (g) during the adsorption process was recorded continuously until equilibrium. The water adsorption capacity of the MIL-101(Cr) sorbent (in ***g_water_*** ***g_coating_*^-1^**) is calculated as:

 (41)

Meanwhile, the water adsorption of the MIL-101(Cr) based on the mass of pure sorbent (in ***g_water_*** ***g_sorben_****_t_*^-1^) is calculated as:

 (42)

where ***m_binder_***, ***m_MOF_*** are the mass of the binder and the pure sorbent in the MIL-101(Cr) coating (g), respectively. This parameter can be used to indicate the possible influence of the binder on the original adsorption capacity of the sorbent.

To calculate the radiative power of an object transferring heat to space, it’s essential to consider the thermal energy the object radiates and the temperature of the space environment. Key factors include the object's temperature, surface properties, radiation area, and the background temperature of space.

For an object radiating heat into space, the Stefan-Boltzmann law can be used, considering the object's temperature and emissivity. The formula is:

 (43)

where: *P* is the radiative power of the object into space (in watts, W); ϵ is the emissivity of the object, with values between 0 and 1 (1 represents a perfect black body); 𝜎 is the Stefan-Boltzmann constant (5.67×10^−8^ W/m ^2^·K ^4^); 𝐴 is the radiative surface area of the object (in square meters, m²); 𝑇 is the surface temperature of the object (in Kelvin, K); *T_∞_* is the background temperature of space, typically around 2.7 K (the temperature of cosmic microwave background radiation).

For the hybrid PTC-TEG-MOF system, during the daytime, compared with the traditional air-cooled TEG design, the heat consumed by the MIL-101(Cr) module during the parsing process can reduce the cold end temperature of the TEG module. For the nighttime hybrid system, compared with the reported TEG module design based on natural cooling, on the one hand, the heat released by the MIL-101(Cr) module during the adsorption process will increase the hot end temperature of the TEG module, and on the other hand, the photothermal composite material reduces the cold end temperature through radiative cooling.

The proposed synergistic thermal effect caused by temperature difference was a field that was rarely studied at present. The novel system not only improved the power density of the TEG module and accelerated the capture and degradation of formaldehyde, but also achieved advanced 24-hour power generation without energy storage compared with the traditional cogeneration system.

**Supplementary Note 6.**

**Seasonal analysis**

**1.Indoor Humidity Increase During Daytime and Air Emission Design:**

In this system, the air first passes through the MOF (Metal-Organic Framework) layer, where it begins to decompose, and water vapor is released, which causes a slight increase in humidity. However, after passing through the MOF layer, the air enters the photothermal catalytic layer, where the heat generated by the catalyst heats the air. This heating process ensures that the water vapor in the air remains within a controlled relative humidity range, preventing the indoor humidity from becoming too high. Therefore, although water vapor is generated during the photocatalytic reaction, the system can effectively regulate humidity by passing the air through these two layers, ensuring that indoor humidity does not increase excessively and avoiding air quality issues caused by excess moisture.

**2.Adaptability to Seasonal Variations:**

The performance of the system varies across different seasons. In summer, higher daytime temperatures lead to an increase in indoor heat load, which may pose challenges to the system’s energy conversion and air purification efficiency. However, the system uses Phase Change Materials (PCMs) to effectively manage temperature fluctuations and store heat, helping to mitigate this issue. Specifically, during summer days, the heat generated by the photocatalytic reaction is stored in the PCM, and at night, this heat is released to assist in power generation, maintain temperature balance, and reduce the need for air conditioning, thus lowering electricity consumption. In winter, when indoor temperatures are lower, the efficiency of the thermocatalytic reaction and power generation modules might be impacted. However, the system’s heat storage capability allows energy to be stored during the day and released at night, ensuring continuous power supply. Moreover, at low temperatures, the reaction efficiency remains relatively high. Therefore, the system performs well in winter, particularly at night, as the PCM module helps to enhance thermoelectric generation efficiency.

In conclusion, although seasonal variations may affect the system’s heat load and power generation capacity, the system’s design, which incorporates coordinated heat storage, thermocatalysis, and thermoelectric generation modules, allows it to effectively adapt to changes between summer and winter. It ensures robust performance in different environmental conditions.

**Supplementary Note 7.**

**Hydroscopic material**

In this study, we selected MIL-101(Cr)/CF as the solid dehumidification material due to its high-water absorption rate, fast water adsorption kinetics, and suitable adsorption/desorption temperatures for outdoor atmospheric applications. Additionally, due to the low thermal conductivity of MIL-101(Cr)/CF, we applied the in-situ impregnation method to coat MIL-101(Cr) powder onto copper foam (CF), thereby preparing MIL-101(Cr)/CF composites as moisture adsorbents. The adsorption experiments were conducted in a constant temperature and humidity chamber, with data recorded using a high-precision electronic balance. Under constant temperature conditions, the equilibrium water adsorption capacity of MIL-101(Cr)/CF increased with increasing relative humidity (30%, 40%, 60%, and 80% RH). The water adsorption isotherm indicates that MIL-101(Cr)/CF exhibits ideal and fast water adsorption properties at 20 °C. The water adsorption performance at different temperatures and relative humidity demonstrates its flexible adaptability. Moreover, multiple adsorption/desorption cycle tests were performed on the MIL-101(Cr)/CF composites, showing an adsorption capacity of approximately 0.925 g·g⁻¹, indicating excellent cyclic stability.

**Supplementary Note 8.**

**Method**

**Ⅰ. Detailed Policy Analysis**

**1. China: Policy Integration and CSP+PV Strategy**

**(1)** China’s Carbon Neutrality Strategy:

China has set the goals of "carbon peak and carbon neutrality," with the clean energy transition being a key pathway to achieve these targets. The country's "14th Five-Year Plan" and "Carbon Peak Action Plan Before 2030" clearly outline key directions for promoting solar energy utilization, particularly the development of solar thermal technology as an important component of low-carbon buildings. This closely aligns with the direction of our research and can provide technical support for China's carbon neutrality goals.

**(2)** National Support for Solar Thermal Utilization in Buildings and Green Buildings:

National policies strongly support the application of solar thermal technologies, especially in the fields of green buildings and building energy efficiency. China's green building standards and solar building integration policies encourage the widespread use of solar thermal technologies in buildings. Our research aligns with these policies, particularly in building integration and thermal energy management, offering effective technical support for energy-saving and emissions reduction in green buildings.

**(3)** Data from 34 Provinces and Cities Nationwide Proving the Applicability of Our Technology on a National Scale:

This study analyzed data from 34 provinces and cities across the country, demonstrating the feasibility of applying our proposed technology nationwide. These data show the solar thermal resource potential and the adaptability of the technology in different regions, providing empirical support for the widespread promotion and application of the technology, in line with national policies aiming at large-scale utilization of solar and renewable energy.

**(4)** The Proposed PTC-TEG-MOF System Supports Distributed Renewable Energy Production and Indoor Air Quality Improvement, Aligning with National Strategies: Solar Building Integration and Technological Innovation in Public Health:

The proposed PTC-TEG-MOF system not only supports the production of distributed renewable energy but also effectively improves indoor air quality, aligning with China's strategic goals for "solar building integration." This system, through solar thermal-electric conversion technology and distributed energy management, drives technological innovation for green buildings and sustainable urban development. It also strongly aligns with national strategies for technological innovation in public health, especially in enhancing indoor air quality and building energy efficiency.

**2. United States: Policy Support and Technological Innovation**

The U.S. government strongly supports the development of solar thermal technology through policies such as the Federal Investment Tax Credit (ITC), particularly in the areas of building energy efficiency and hot water supply. Research projects funded by the U.S. Department of Energy (DOE) have driven innovation in thermal technology, particularly in improving system efficiency and reducing costs. U.S. policies support the integration of solar thermal systems with buildings, encouraging their installation in residential and commercial buildings. Through long-term market incentives such as Power Purchase Agreements (PPAs) and tax benefits, the U.S. is committed to promoting the widespread adoption of solar thermal technology to achieve green energy goals.

Reference websites: [https://www.irena.org/, https://www.energy.gov/eere/solar/solar-energy-technologies-office](https://www.irena.org/,%20https://www.energy.gov/eere/solar/solar-energy-technologies-office)

**3.Germany: Transition and Solar Thermal Applications**

Germany, as part of its "Energy Transition" strategy, not only focuses on photovoltaics and wind energy but also actively promotes the use of solar thermal technology. The German government encourages the integration of solar thermal systems in buildings through green building standards, financial subsidies, and support for their integration into energy-efficient building designs. Solar thermal technology is widely applied in building hot water and heating systems. By providing fixed feed-in tariffs and gradually reducing financial support, Germany encourages market competition, enhancing the commercialization of solar thermal technology. The country's policy framework emphasizes technological innovation and building energy.

Reference websites: https://www.bmwk.de/Navigation/EN/Home/home.html

**4. Spain: Promoting Solar Thermal Applications**

Spain has been a major advocate for the use of solar thermal technology, particularly in residential and commercial building integration. The government supports the installation of solar thermal systems through financial subsidies and tax incentives, especially for heating and hot water systems. The Spanish government also encourages the integration of solar thermal systems with building designs and energy management systems to enhance the energy efficiency and sustainability of buildings. The successful application of solar thermal technology in Spain provides valuable lessons for other countries, particularly in the innovative practices of integrating solar energy with buildings.

Reference websites: https://www.irena.org/, https://www.mimit.gov.it/index.php/en/

**5.Italy: Solar Thermal and Building Integration**

Italy has been at the forefront of applying solar thermal technology, especially in the integration of solar thermal systems with buildings. The government promotes the widespread adoption of solar thermal technology in buildings through green building policies and tax incentives. Italy's "Building Integrated Solar Thermal" policy has encouraged the deployment of thermal systems, especially in building heating and hot water supply. The country has introduced incentives to encourage buildings to adopt solar thermal systems, improving energy efficiency and reducing energy consumption. The use of solar thermal technology not only helps achieve sustainable building goals but also contributes to the reduction of greenhouse gas emissions.

Reference websites: https://www.miteco.gob.es/en.html,

**6.India: Promoting Solar Thermal Technology**

The Indian government has set ambitious renewable energy goals, particularly in the solar sector. The National Solar Mission aims to achieve a renewable energy installed capacity of 500GW by 2030, with solar thermal technology playing a key role in this strategy. The government promotes the adoption of solar thermal systems through subsidy policies and long-term Power Purchase Agreements (PPAs), particularly in rural and remote areas. India places a strong emphasis on the application of solar thermal technology in hot water and heating systems, while also supporting large-scale solar thermal power projects. These efforts are aimed at addressing energy shortages and reducing dependence on coal.

Reference websites: https://www.iea.org/reports/india-energy-outlook

**7.South Korea: Integration of Solar Thermal Technology with Green Buildings**

The South Korean government has integrated solar thermal technology with green building policies and actively promotes the installation of solar hot water systems and heating systems in new and renovated buildings. The government supports these efforts through financial subsidies and technical assistance, encouraging businesses and residents to integrate solar thermal technology into buildings. South Korea’s solar thermal projects go beyond hot water and heating systems, also incorporating air quality management and smart building technologies, which enhance building energy efficiency and improve indoor environmental quality. These policies and technological innovations align with South Korea's goals of sustainable urban development and public health.

Reference websites: https://www.irena.org/, https://english.motie.go.kr/

**8.United Kingdom: Clean Energy Transition and the Promotion of Solar Thermal Energy**

The UK government has developed a clear strategy to promote the clean energy transition, with solar thermal technology playing a key role in this effort. According to the UK’s "2050 Carbon Neutrality Target," the government actively supports the research, development, and application of solar thermal technologies, especially in building energy efficiency and heating systems. The UK encourages the integration of solar thermal technology through fiscal subsidies, tax incentives, and green building standards, particularly in newly built and renovated residential and commercial buildings. The widespread application of solar thermal systems not only helps reduce building energy consumption but also promotes the development of sustainable buildings. The government is also advancing smart building technologies to further enhance the efficiency and performance of solar thermal systems, driving the construction of green buildings and low-carbon cities.

Reference websites: https://www.gov.uk/government/organisations/department-for-business-energy-and-industrial-strategy, https://www.irena.org/

**9. France: Solar Thermal Technology and the Enhancement of Energy Efficiency**

The French government places great importance on solar thermal technology, particularly in improving building energy efficiency. As one of the European leaders in green building advocacy, France has established stringent building energy efficiency standards and encourages the use of solar thermal systems in residential, commercial, and public buildings. Solar thermal technology is widely applied in hot water supply and heating systems, and the government further promotes its adoption through green building incentives, financial subsidies, and low-interest loans. In addition, local governments offer solar thermal subsidy programs, especially in small cities and rural areas, to assist these regions in utilizing clean energy. The development of solar thermal technology in France not only helps reduce carbon emissions but also contributes to the growth of the green economy, aligning with the country’s long-term sustainability goals.

Reference websites: <https://www.ecologie.gouv.fr/>, https://www.irena.org/

**Supplementary Note 9.**

Sensitivity analysis typically involves calculating the response of the output variable relative to the changes in the input variable when one variable is altered. In this case, we can quantitatively analyze the effect of the independent variable on the dependent variable by using the percentage change rate. We can calculate this using the following formula:

$Sensitivity=\frac{\text{Δ}Y}{\text{Δ}X}=\frac{Y_{2}-Y_{1}}{X_{2}-X_{1}}$ (44)

**(1) Analysis of the Impact of Temperature Difference on System Power Density**

Through sensitivity analysis, we observe that in the low-temperature difference range (2°C-5°C), the temperature difference has a significant impact on power density, with a rapid increase in power density and high sensitivity. Therefore, under low temperature differences, the system responds more sensitively, leading to higher power density output. In the moderate temperature difference range (5°C-9°C), the system's sensitivity gradually decreases, and the increase in power density slows down. The effect of temperature difference on power density becomes less significant. In the high temperature difference range (9°C-13°C), as the temperature difference increases, the growth of power density levels off, and the system's response to temperature difference changes weakens.

The sensitivity analysis indicates that temperature difference plays a significant role in increasing power density in the low temperature difference range, but in higher temperature differences, the growth of power density gradually saturates. If higher power density output is desired in the high temperature difference range, further optimization of the system design or the consideration of additional strategies to increase power may be needed. If the system is used in environments with small temperature differences, the system's power density will experience a significant boost and work efficiently. However, in environments with large temperature variations, it may be necessary to enhance the system's responsiveness to temperature changes or to improve system performance in the high temperature difference range through other techniques (such as increasing the temperature difference source or improving thermoelectric conversion efficiency).

**(2) Analysis of the Impact of Irradiation Intensity on Purification Performance**

From the data, it is evident that there is a positive correlation between irradiation intensity and single-pass rate. However, as the irradiation intensity increases, the increment in the single-pass rate gradually diminishes, indicating that the system's sensitivity to irradiation intensity changes across different intensity ranges.

In the low irradiation intensity range (0 W/m^2^to 300 W/m^2^), the increase in the single-pass rate is quite significant. For example, when the irradiation intensity increases from 0 W/m^2^ to 100 W/m^2^, the single-pass rate increases by 13.71%, from 62.21% to 70.34%. Similarly, from 100 W/m^2^ to 200 W/m^2^, the increase is 8.13%, showing a strong response. In this range, the system is highly sensitive to changes in irradiation intensity, and the single-pass rate increases noticeably. As the irradiation intensity gradually increases to 600 W/m^2^, the increase in the single-pass rate starts to diminish. For instance, from 300 W/m^2^ to 400 W/m^2^, the increase is 4.04%, and from 400 W/m^2^ to 500 W/m^2^, the increase is 2.54%. This suggests that with the increasing irradiation intensity, the system's response to irradiation intensity weakens, and the increase in the single-pass rate becomes smaller in this range. In the higher irradiation intensity range (600 W/m^2^ to 1000 W/m^2^), the increment in the single-pass rate becomes very small. From 600 W/m² to 700 W/m², the increase in the single-pass rate is 0.7%; from 700 W/m^2^ to 800 W/m^2^, it increases by 0.78%; and from 900 W/m^2^ to 1000 W/m^2^, the increase is 3%. This gradual increase indicates that the system's sensitivity weakens as the irradiation intensity increases, entering a state of saturation.

Based on this sensitivity analysis, if the system is used in environments with low irradiation intensity, the system will respond more sensitively to changes in irradiation intensity, leading to a significant improvement in the single-pass rate. Therefore, the system will perform better in low irradiation environments. However, in environments with higher irradiation intensity, the improvement in the single-pass rate becomes limited. This may suggest that after a certain threshold of irradiation intensity, the system's efficiency reaches saturation. In such cases, it would be necessary to consider optimizing other factors or adding additional functionalities to enhance the system's overall performance under high irradiation conditions.

**(3) Analysis of the Impact of Temperature on Purification Performance**

From the data, it can be observed that there is a positive correlation between temperature and single-pass rate. As the temperature increases, the single-pass rate continues to rise, indicating that temperature has a significant impact on the system's performance.

In the lower temperature range (20°C to 40°C), the increase in the single-pass rate is substantial. For instance, from 20°C to 30°C, the single-pass rate increases by 4.81%, and from 30°C to 40°C, it increases by 6.29%. In this range, the rise in temperature has a noticeable effect on the single-pass rate, and the system responds sensitively to temperature changes. However, as the temperature continues to increase, from 40°C to 50°C, the increase in the single-pass rate is 3.33%, and from 50°C to 60°C, the increase is 4.9%. Although the single-pass rate still increases, the rate of increase begins to slow down. Compared to the lower temperature range, the system's sensitivity to temperature changes diminishes, showing a reduction in responsiveness. In the higher temperature range (60°C to 80°C), the effect of temperature on the single-pass rate starts to stabilize. Specifically, from 60°C to 70°C, the single-pass rate increases by 8.1%, and from 70°C to 80°C, it increases by 5.29%. While temperature still has a significant impact on the single-pass rate in this range, the increment begins to slow down, indicating that the system's sensitivity to temperature decreases in high-temperature environments.

The sensitivity analysis shows that temperature changes have a more significant effect on the system in the low and medium temperature ranges, while at high temperatures, the system's response to temperature changes tends to reach saturation.

**(4) Analysis of the Impact of Relative Humidity Variation on MOF Moisture Absorption Performance**

The impact of relative humidity on the moisture absorption performance of MOF is not linear but gradually weakens as the humidity increases. Specifically, in the low humidity range (20%-30% RH and 30%-40% RH), the rate of increase in the moisture absorption per gram of MOF is relatively high (0.011 g/%RH and 0.05 g/%RH). As the humidity increases, the system’s moisture absorption starts to approach saturation, with the change in moisture absorption becoming smaller between 50%-60% RH and 60%-70% RH, and the increase in the amount of absorbed water gradually slows down. When the humidity is near saturation (70%-80%RH), the moisture absorption becomes almost stable, with the increase in the moisture absorption per gram of MOF being only 0.003 g/%RH.

In the high humidity range (above 50%RH), the increase in the moisture absorption of MOF material becomes smaller. This suggests that within this humidity range, the material’s moisture absorption performance may reach a saturation point, and further increases in humidity have a diminished effect on the dehumidification performance. In the low humidity range (20%-40% RH), the moisture absorption of MOF material increases significantly, indicating high sensitivity. This range represents the most effective moisture absorption range for MOF materials.

The sensitivity analysis indicates that if MOF material is applied in environments with low humidity, its dehumidification performance will be more significant. In high humidity environments, the moisture absorption performance of MOF material gradually weakens, and it may require improvements in the material’s absorption capacity or other methods to enhance the dehumidification efficiency.

**List of Figures 1-39**

**Supplementary Figure 1.** The preparation process and molecular structure of the material.

**Supplementary Figure 2.** EPR patterns of Mn_7_Co_3_Ce_1_O_x_, and related elements (Co, Mn, and Ce).

**Supplementary Figure 3.** The effect of surface formaldehyde concentration on apparent reaction rate under photothermal coupling catalytic mode at different temperatures (reaction condition: irradiation intensity of 0 mW/cm^2^), b-c) Linear fitting of ln(k_1_) or ln(k_2_) with 1/T using Arrhenius equation (reaction condition: irradiation intensity 0 mW/cm^2^); d) The effect of surface formaldehyde concentration on apparent reaction rate under photothermal coupling catalytic mode at different temperatures (reaction condition: irradiation intensity of 1.1 mW/cm^2^), e-f) Linear fitting of ln(k_1_) or ln(k_1_) with 1/T using Arrhenius equation (reaction condition: irradiation intensity 1.1 mW/cm^2^).

**Supplementary Figure 4.** The reusability of Mn_7_Co_3_Ce_1_O_x_ material.

**Supplementary Figure 5.** (a) HAADF-STEM image and (b-f) corresponding element (C, O, Mn, Co, Ce) mapping of Mn_7_Co_3_Ce_1_O_x_.

**Supplementary Figure 6**. Excitation wavelength of Mn_7_Co_3_Ce_1_O_x_ based on Planck's formula(a), and the single-pass rate of Mn_7_Co_3_Ce_1_O_x_ under thermocatalytic conditions(b).

**Supplementary Figure 7.** Water adsorption isotherms of MIL-101(Cr) with typical S-shaped characteristics and high-water uptake at 20°C.

**Supplementary Figure 8.** Water uptake of MIL-101(Cr)/CF composite samples at different temperature and RH.

**Supplementary Figure 9.** Cycling stability of moisture absorption-desorption of MIL-101(Cr)/CF (absorption at 20°C and 80% RH, desorption at 70 °C and 30% RH).

**Supplementary Figure 10.** Long-term cycling performance of MIL-101(Cr)/CF in water adsorption.

**Supplementary Figure 11.** The temperature evaluations of PTC and MOF in the hybrid PTC-TEG-MOF device.

**Supplementary Figure 12.** The power density evaluations of the TEG module.

**Supplementary Figure 13.** The conversion efficiency of the TEG module.

**Supplementary Figure 14.** The relative humidity of the hybrid PTC-TEG-MOF device.

**Supplementary Figure 15.** The temperature change of air inlet and outlet.

**Supplementary Figure 16.** The temperature evaluations of PTC and MOF in the hybrid PTC-TEG-MOF device.

**Supplementary Figure 17.** The power density evaluations of the TEG module in the hybrid devices with and without MOF.

**Supplementary Figure 18.** The power density evaluations of the TEG module.

**Supplementary Figure 19.** The conversion efficiency evaluations of the TEG module.

**Supplementary Figure 20.** The energy consumption analysis of air conditioning with or without PTC-TEG-MOF hybrid system.

**Supplementary Figure 21.** The CADR values.

**Supplementary Figure 22.** The temperature evaluations of the TEG module.

**Supplementary Figure 23.** The power density evaluations of the TEG module.

**Supplementary Figure 24.** The conversion efficiency evaluations of the TEG module.

**Supplementary Figure 25.** The non-purification-desorption mode(a), and non-purification-dehumidification mode(b).

**Supplementary Figure 26.** The comparison of simulated and experimental results.

**Supplementary Figure 27.** The comparison of thermoelectric power results from different researchers: a: purification performance; b: power generation performance; c: absorptivity.

**Supplementary Figure 28.** The preparation process of MnCo_3_Ce_1_O_x_ material.

**Supplementary Figure 29.** The preparation process of MIL-101 (Cr) material.

**Supplementary Figure 30.** The flow chart(a) and physical layout(b) of steady-state experiments during daytime and nighttime.

**Supplementary Figure 31.** The uncertainty of the experiment results.

**Supplementary Figure 32**. The comparison of thermoelectric power results from different researchers

**Supplementary Figure 33.** Spectral properties of Mn_7_Co_3_Ce_1_O_x_ materials.

**Supplementary Figure 34.** The influence of different factors.


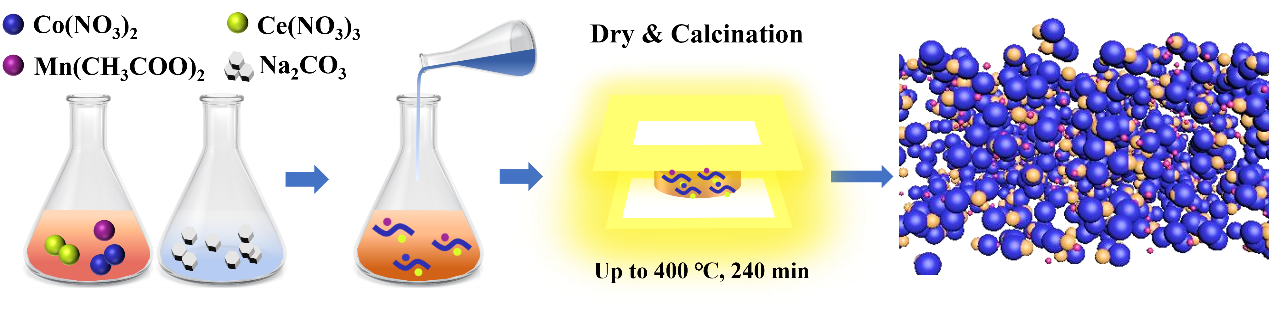


**Supplementary Figure 1.** The preparation process and molecular structure of the material.

**Supplementary Figure 2.** EPR patterns of Mn_7_Co_3_Ce_1_O_x_, and related elements (Co, Mn, and Ce).


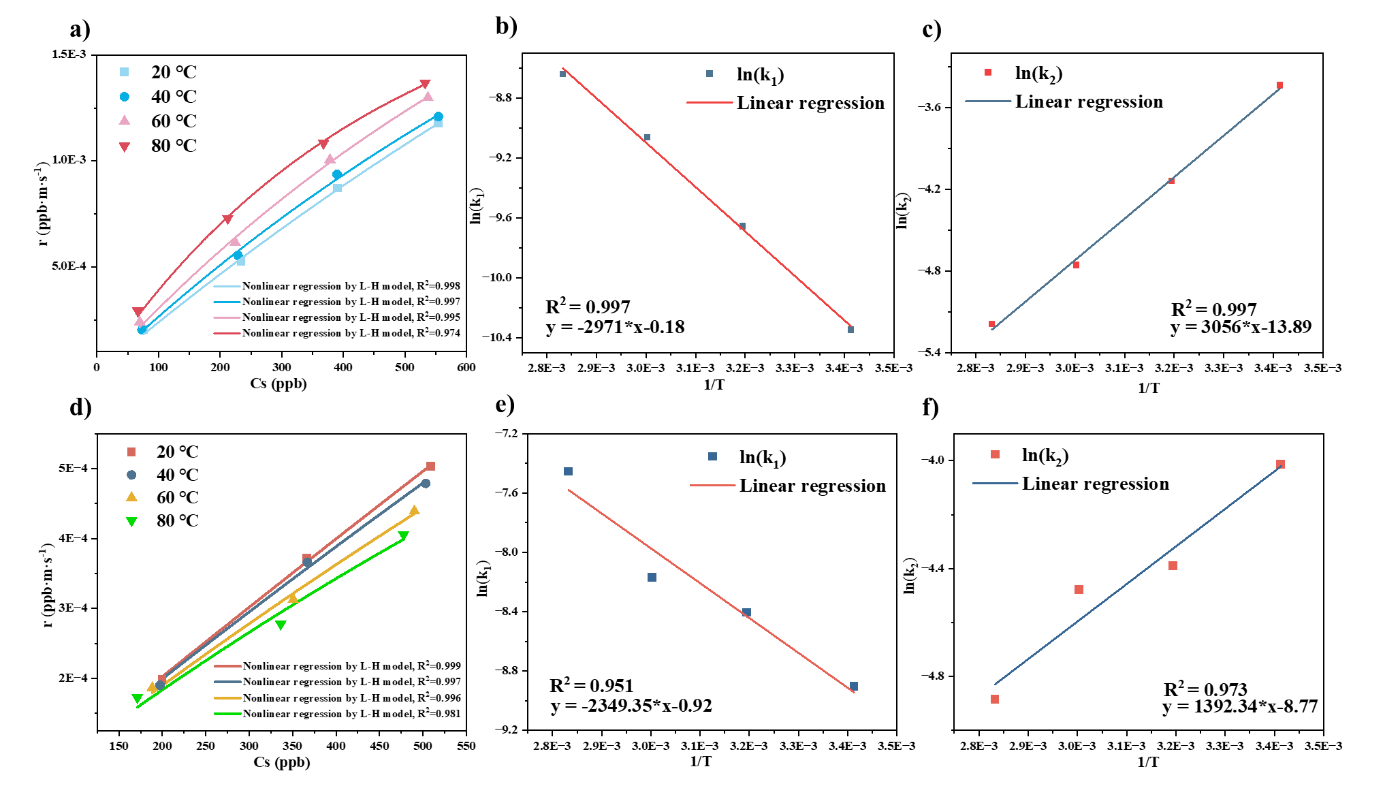


**Supplementary Figure 3.** The effect of surface formaldehyde concentration on apparent reaction rate under photothermal coupling catalytic mode at different temperatures (reaction condition: irradiation intensity of 0 mW/cm^2^), b-c) Linear fitting of ln(k_1_) or ln(k_2_) with 1/T using Arrhenius equation (reaction condition: irradiation intensity 0 mW/cm^2^); d) The effect of surface formaldehyde concentration on apparent reaction rate under photothermal coupling catalytic mode at different temperatures (reaction condition: irradiation intensity of 1.1 mW/cm^2^), e-f) Linear fitting of ln(k_1_) or ln(k_1_) with 1/T using Arrhenius equation (reaction condition: irradiation intensity 1.1 mW/cm^2^).

**Supplementary Figure 4.** The reusability of Mn7Co3Ce1Ox material.


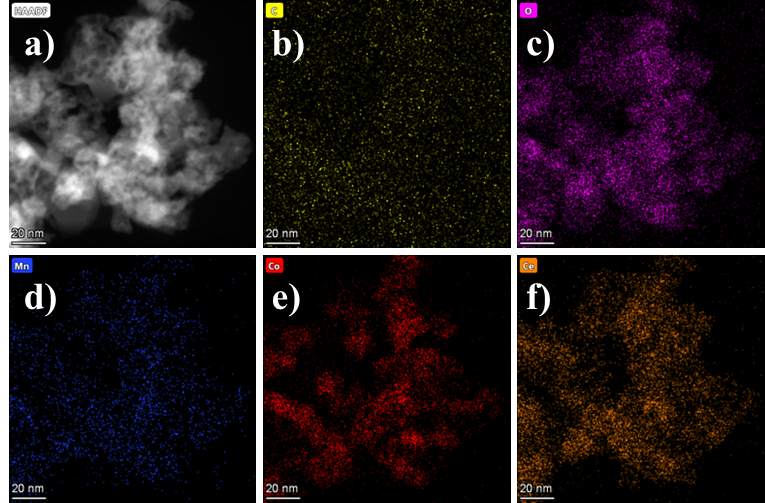


**Supplementary Figure 5.** (a) HAADF-STEM image and (b-f) corresponding element (C, O, Mn, Co, Ce) mapping of Mn_7_Co_3_Ce_1_O_x_.


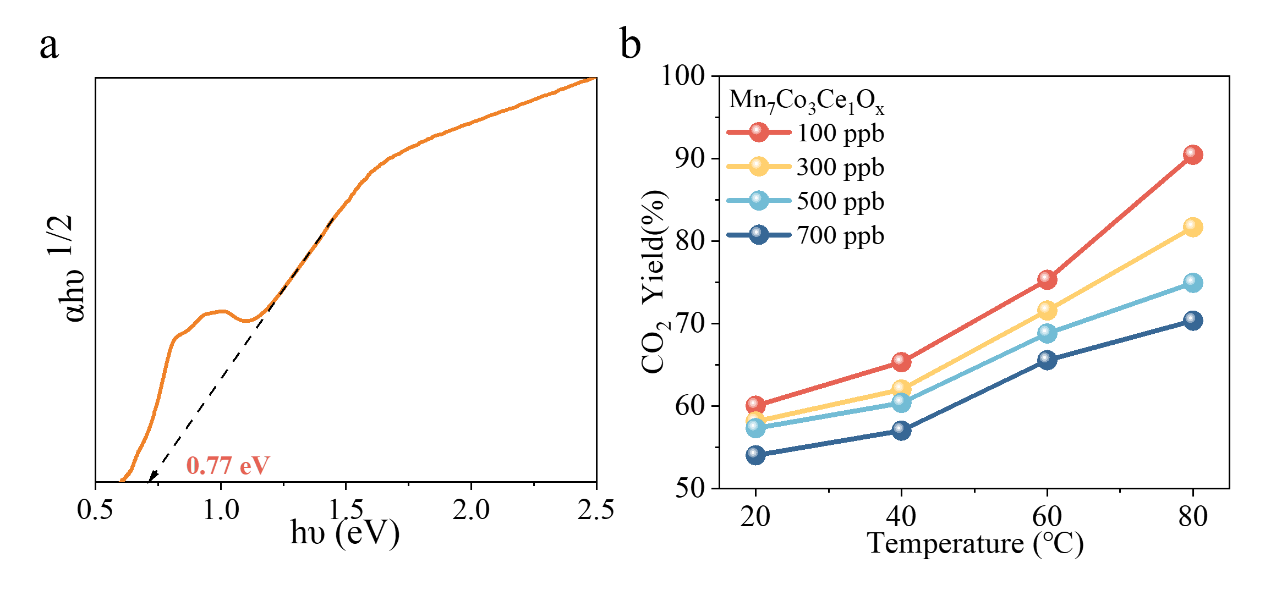


**Supplementary Figure 6.** Excitation wavelength of Mn_7_Co_3_Ce_1_O_x_ based on Planck's formula(a), and the single-pass rate of Mn_7_Co_3_Ce_1_O_x_ under thermocatalytic conditions(b).

**Supplementary Figure 7.** Water adsorption isotherms of MIL-101(Cr) with typical S-shaped characteristics and high-water uptake at 20°C.

**Supplementary Figure 8.** Water uptake of MIL-101(Cr)/CF composite samples at different temperature and RH.

**Supplementary Figure 9.** Cycling stability of moisture absorption-desorption of MIL-101(Cr)/CF (absorption at 20°C and 80% RH, desorption at 70 °C and 30% RH).

**Supplementary Figure 10.** Long-term cycling performance of MIL-101(Cr)/CF in water adsorption.

**Supplementary Figure 11.** The temperature evaluations of PTC and MOF in the hybrid PTC-TEG-MOF device.


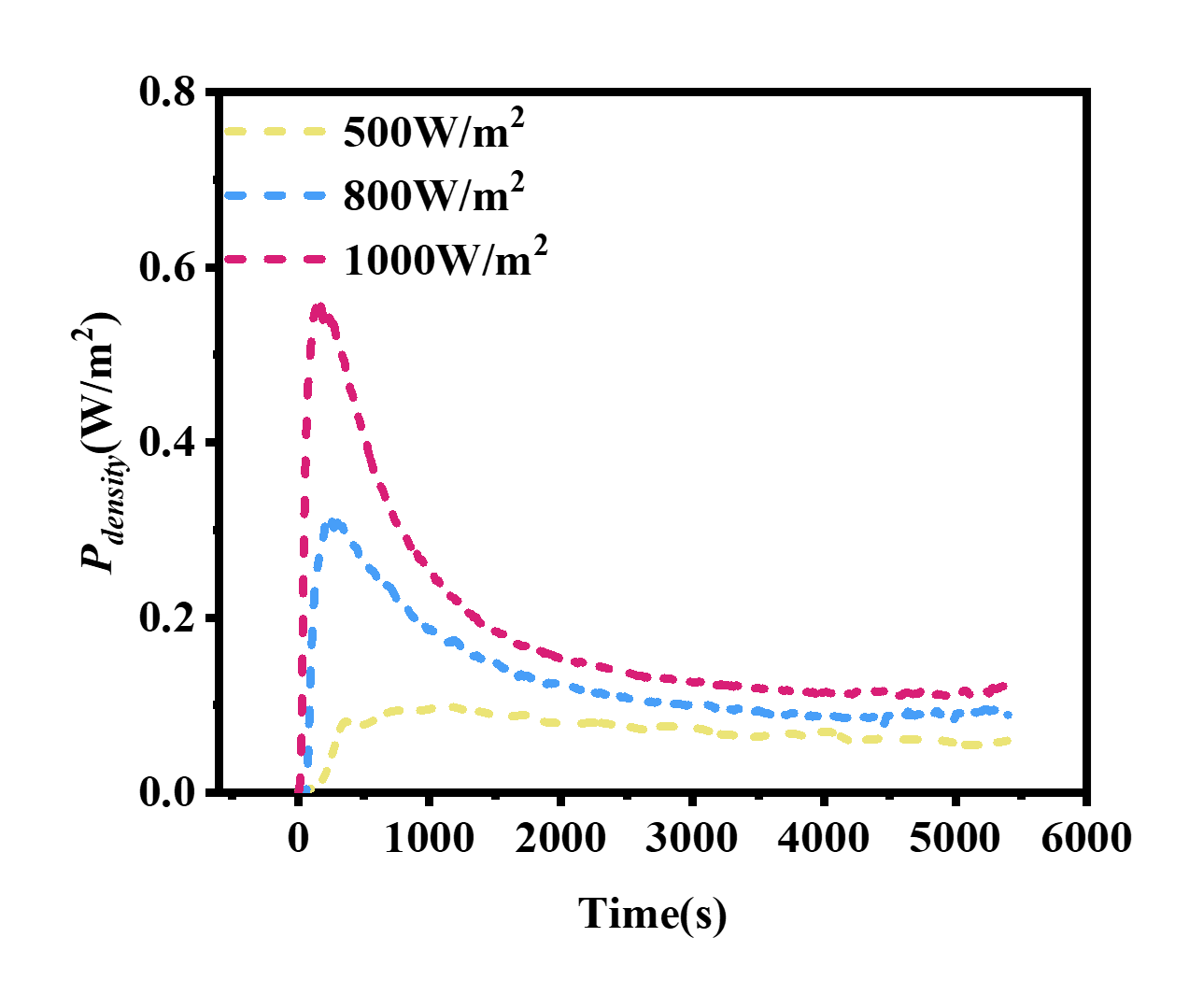


**Supplementary Figure 12.** The power density evaluations of the TEG module.

**Supplementary Figure 13.** The conversion efficiency of the TEG module.

**Supplementary Figure 14.** The relative humidity of the hybrid PTC-TEG-MOF device.

**Supplementary Figure 15.** The temperature change of air inlet and outlet.

**Supplementary Figure 16.** The temperature evaluations of PTC and MOF in the hybrid PTC-TEG-MOF device.

**Supplementary Figure 17.** The power density evaluations of the TEG module in the hybrid devices with and without MOF.

**Supplementary Figure 18.** The power density evaluations of the TEG module.

**Supplementary Figure 19.** The conversion efficiency evaluations of the TEG module.

**Supplementary Figure 20.** The energy consumption analysis of air conditioning with or without PTC-TEG-MOF hybrid system.

**Supplementary Figure 21.** The CADR values.

**Supplementary Figure 22.** The temperature evaluations of the TEG module.

**Supplementary Figure 23.** The power density evaluations of the TEG module.

**Supplementary Figure 24.** The conversion efficiency evaluations of the TEG module.


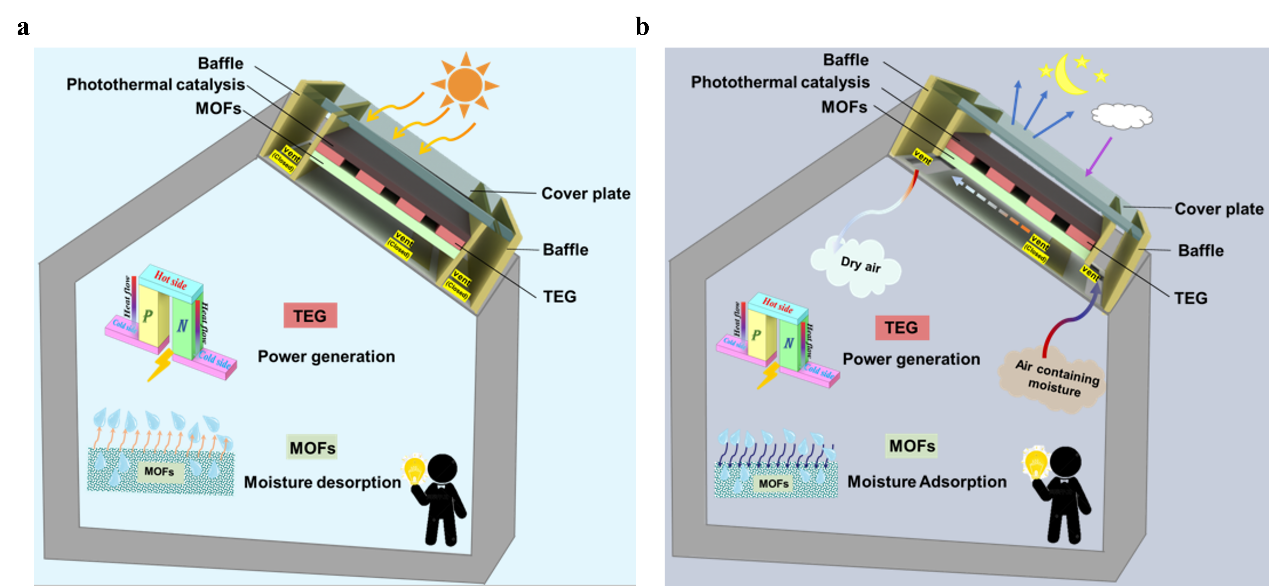


**Supplementary Figure 25.** The non-purification-desorption mode(a), and non-purification-dehumidification mode(b).


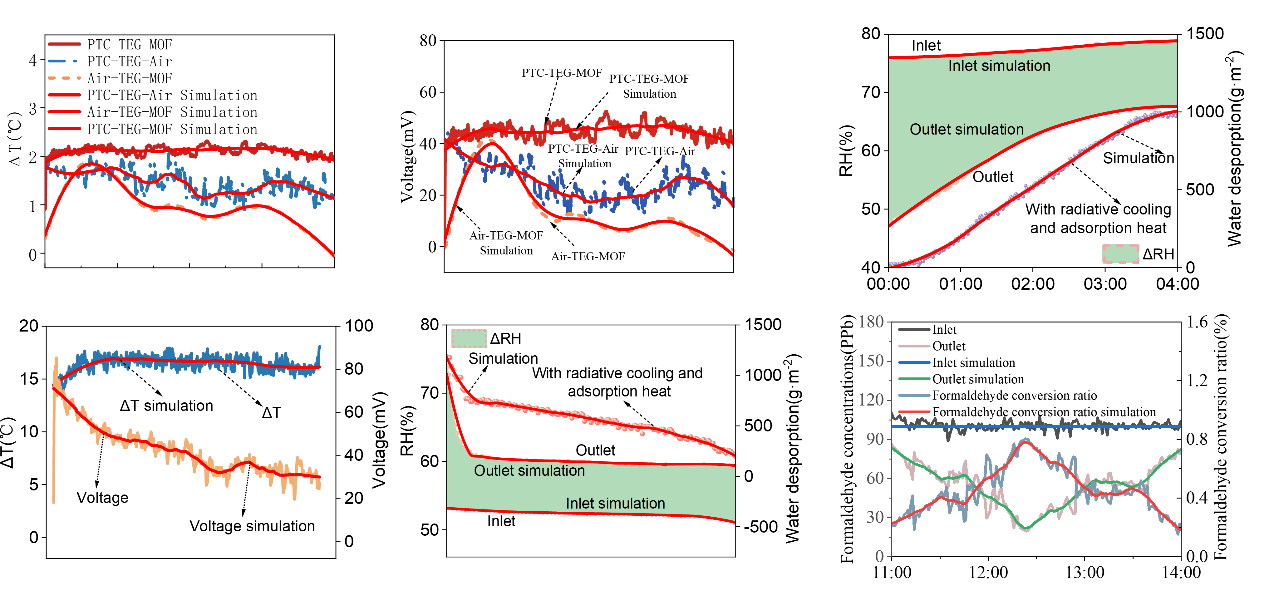


**Supplementary Figure 26.** The comparison of simulated and experimental results.


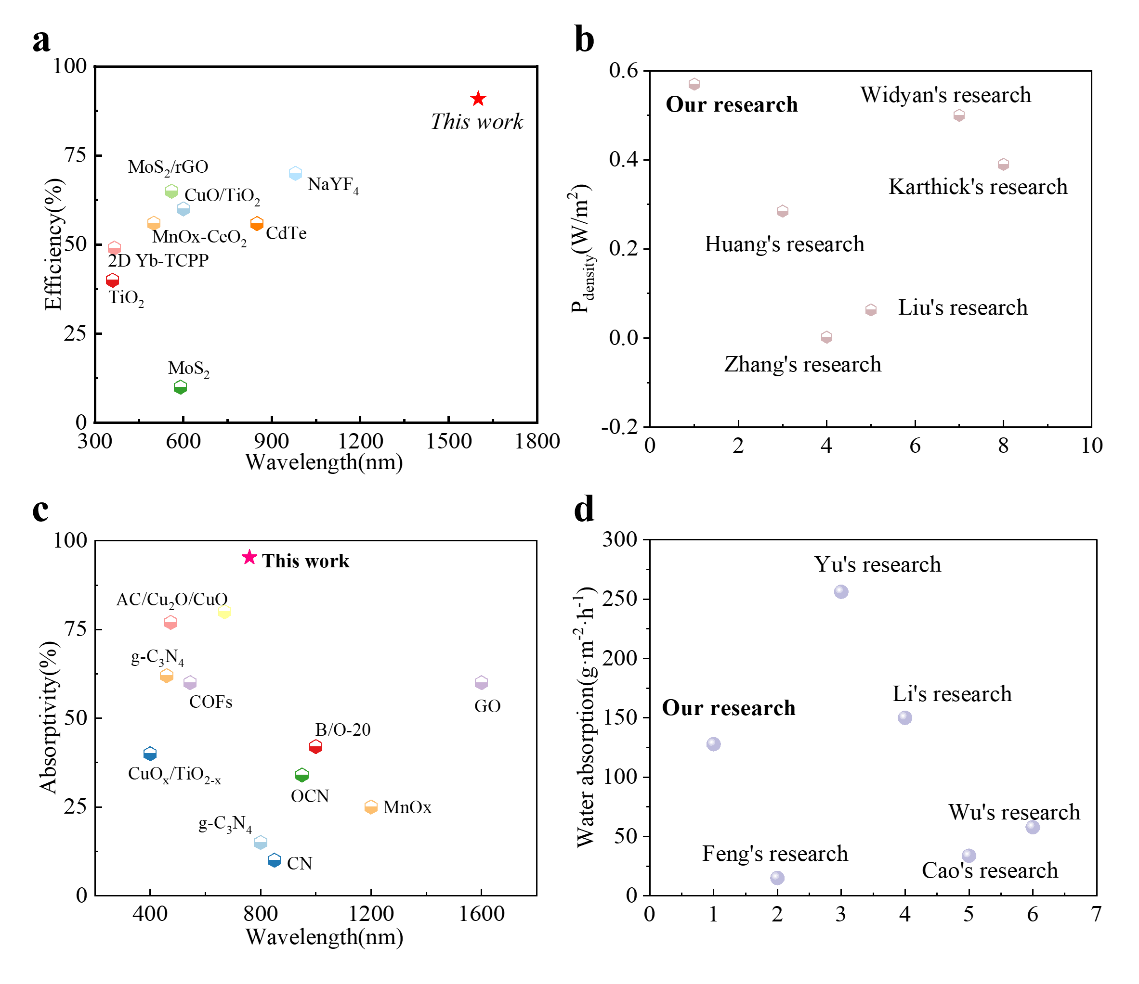


**Supplementary Figure 27.** The comparison of thermoelectric power results from different researchers^(^*^25-32^*^)^ a: purification performance; b: power generation performance; c: absorptivity.

**Supplementary Figure 28.** The preparation process of MnCo_3_Ce_1_O_x_ material.

**Supplementary Figure 29.** The preparation process of MIL-101 (Cr) material.

**Supplementary Figure 30.** The flow chart(a) and physical layout(b) of steady-state experiments during daytime and nighttime.

**Supplementary Figure 31.** The uncertainty of the experiment results.


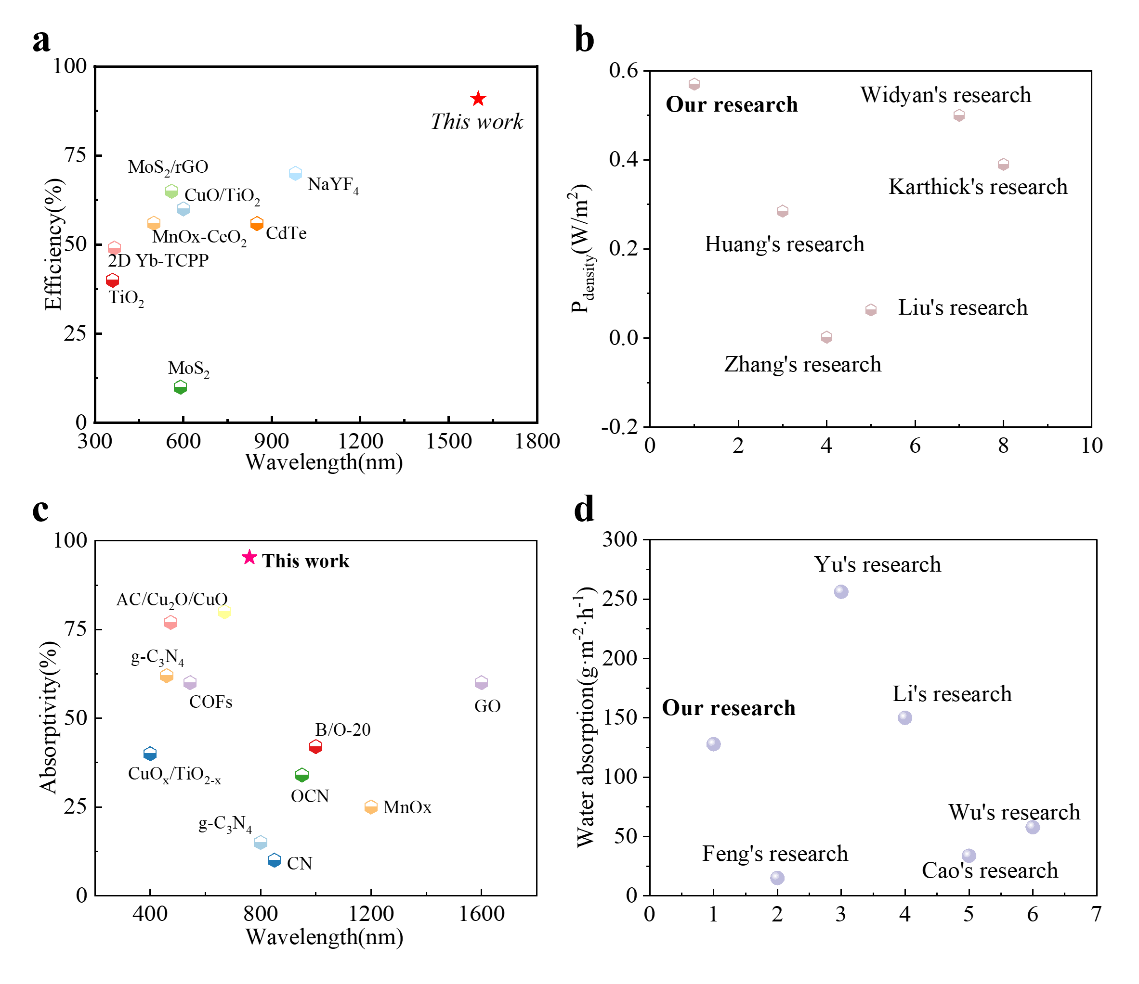


**Supplementary Figure 32**. The comparison of thermoelectric power results from different researchers(*25-32*) a: purification performance; b: power generation performance; c: absorptivity; d: water absorption(*33-36*).

**Supplementary Figure 33.** Spectral properties of Mn_7_Co_3_Ce_1_O_x_ materials.


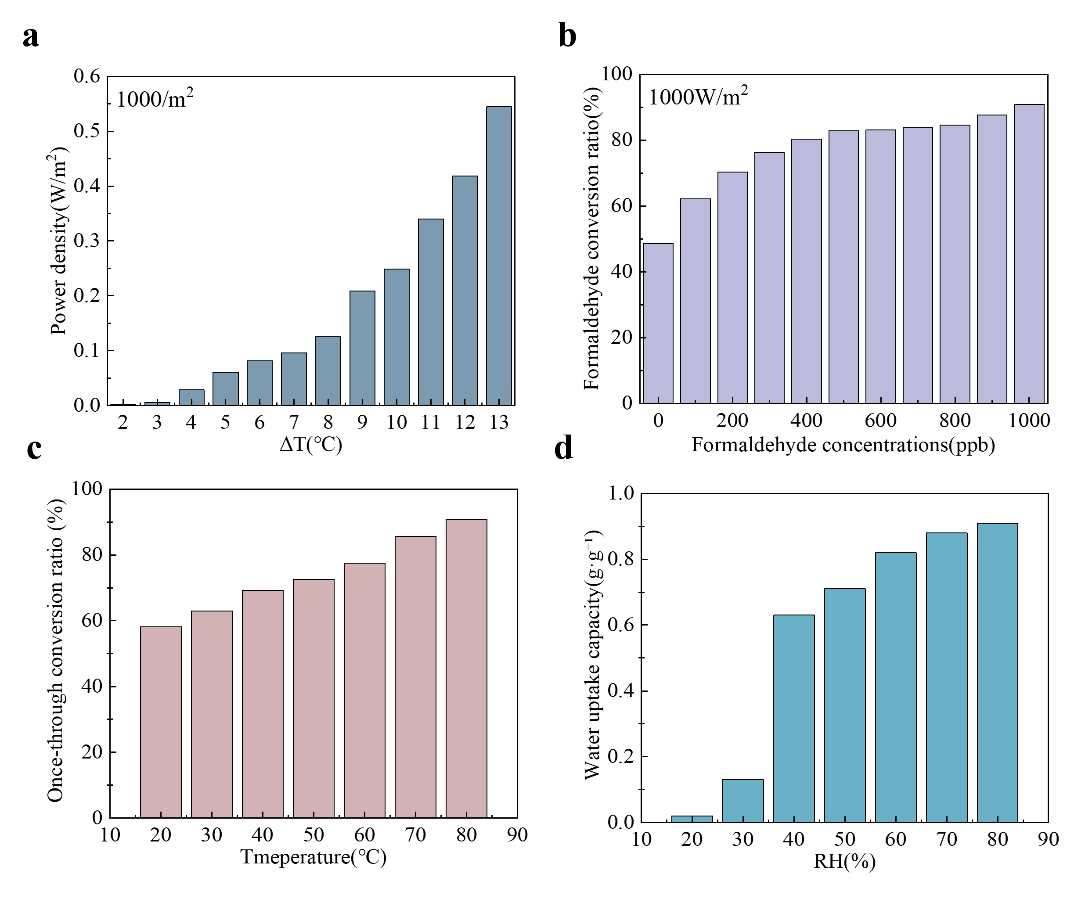


**Supplementary Figure 34.** The influence of different factors.

**Supplementary Table 1.** The physical parameters of each material used in simulation calculation.

| **Material** | **Glass** | **Catalyst layer** | **TEG** |
| --- | --- | --- | --- |
| *ρ*, kg/m^3^ | 1200 | 4000-6000 | 7000 |
| *c_p_*, J/kg∙K | 1900 | 500-1000 | 390 |
| *λ*, W/m∙K | 0.22 | 1-5 | 398 |
| Thickness, m | 0.01 | 0.001 | 0.05 |
| *α* |  | 0.95 | 0.05 |
| Transmittance | 0.93 | 0.01 |  |

**Supplementary Table 2.** Experimental instruments.

| **Apparatus** | **Model** | **Measuring parameter** | **Accuracy** |
| --- | --- | --- | --- |
| Data acquisition instrument | Agilent34972 A | Temperature, Solar radiation intensity | / |
| Thermocouple | K type | Temperature | ±0.5 °C |
| Solar irradiator | TQB-2 | Global solar radiation | ±11.04 |
| Hot-wire anemometer | KANOMAX | Air flow velocity | ±0.01 m/s |
| Electronic balance | STX1202ZH | Mass | ±0.01g |
| Humidity Detector | KST03FOR | Relative humidity | / |
| Formaldehyde detector | HCX400-CH2O | Formaldehyde concentration | 2% |

**Supplementary Table 3.** The fitting results of Arrhenius equation under different irradiation intensities.

| *I*  (mW/cm^2^) | *E*_1_  (kJ·mol^-1^·K^-1^) | *R^2^* | *E*_2_  (kJ·mol^-1^·K^-1^) | *R^2^* |
| --- | --- | --- | --- | --- |
| 0 | 24.69 | 0.997 | -25.39 | 0.997 |
| 1.1 | 19.52 | 0.951 | -11.57 | 0.973 |

**Supplementary References**

1. W. Gu, T. Ma, A. Song, M. Li, L. Shen, Mathematical modelling and performance evaluation of a hybrid photovoltaic-thermoelectric system. *Energy Conversion and Management* **198**, 111800 (2019).

2. T. Ma, W. Gu, L. Shen, M. Li, An improved and comprehensive mathematical model for solar photovoltaic modules under real operating conditions. *Solar Energy* **184**, 292-304 (2019).

3. T. Ma, J. Zhao, Z. Li, Mathematical modelling and sensitivity analysis of solar photovoltaic panel integrated with phase change material. *Applied Energy* **228**, 1147-1158 (2018).

4. B. Yu, N. Li, J. Ji, Performance analysis of a purified Trombe wall with ventilation blinds based on photo-thermal driven purification. *Applied Energy* **255**, (2019).

5. Y. Li, B. Yu, N. J. E. Li, Buildings, The performance analysis of a novel manganese oxide solar low-temperature thermal-catalyst in building multifunctional applications. **297**, 113477 (2023).

6. P. Berdahl, M. Martin, Emissivity of clear skies. *Solar Energy* **32**, 663-664 (1984).

7. T. M. J. Nilsson, G. A. Niklasson, Radiative cooling during the day: simulations and experiments on pigmented polyethylene cover foils. *Solar Energy Materials and Solar Cells* **37**, 93-118 (1995).

8. Z. Shi *et al.*, Maximizing energy generation: A study of radiative cooling-based thermoelectric power devices. *Energy* **274**, 127283 (2023).

9. C. Zhang, C. Shen, Y. Zhang, J. Pu, Feasibility investigation of spectral splitting photovoltaic/thermal systems for domestic space heating. *Renewable Energy* **192**, 231-242 (2022).

10. C. Zhang, C. Shen, Y. Zhang, J. Pu, Feasibility investigation of spectral splitting photovoltaic /thermal systems for domestic space heating. *Renewable Energy* **192**, 231-242 (2022).

11. C. Guo *et al.*, Numerical simulation and experimental validation of tri-functional photovoltaic/thermal solar collector. *Energy* **87**, 470-480 (2015).

12. D. Zhao *et al.*, Radiative sky cooling: Fundamental principles, materials, and applications. *Applied Physics Reviews* **6**, (2019).

13. J. Wang, Z. Zhou, J. Zhao, A method for the steady-state thermal simulation of district heating systems and model parameters calibration. *Energy Conversion and Management* **120**, 294-305 (2016).

14. S. Gupta *et al.*, Foliar smoke-water application improves growth and changes the leaf mineral composition of radish, spinach and turnip seedlings. *South African Journal of Botany* **167**, 527-534 (2024).

15. H. Bahaidarah, A. Subhan, P. Gandhidasan, S. Rehman, Performance evaluation of a PV (photovoltaic) module by back surface water cooling for hot climatic conditions. *Energy* **59**, 445-453 (2013).

16. G. Li *et al.*, A review of solar photovoltaic-thermoelectric hybrid system for electricity generation. *Energy* **158**, 41-58 (2018).

17. S. Lv *et al.*, Research and numerical analysis on performance optimization of photovoltaic-thermoelectric system incorporated with phase change materials. *Energy* **263**, 125850 (2023).

18. Q. Yan, M. G. Kanatzidis, High-performance thermoelectrics and challenges for practical devices. *Nature Materials* **21**, 503-513 (2022).

19. W. G. J. H. M. v. Sark, Feasibility of photovoltaic – Thermoelectric hybrid modules. *Applied Energy* **88**, 2785-2790 (2011).

20. W. Wei *et al.*, A continuous 24-hour power generated PV-TEG-PCM hybrid system enabled by solar diurnal photovoltaic/thermal conversion and nocturnal sky radiative cooling. *Energy Conversion and Management* **321**, 119086 (2024).

21. L. Zhu, T. Ding, M. Gao, C. K. N. Peh, G. W. Ho, Shape Conformal and Thermal Insulative Organic Solar Absorber Sponge for Photothermal Water Evaporation and Thermoelectric Power Generation. *Advanced Energy Materials* **9**, 1900250 (2019).

22. T. Ding *et al.*, Hybrid Photothermal Pyroelectric and Thermogalvanic Generator for Multisituation Low Grade Heat Harvesting. *Advanced Energy Materials* **8**, 1802397 (2018).

23. D. M. Rowe, *CRC handbook of thermoelectrics*. (CRC press, 2018).

24. J. Xiong *et al.*, Technology pathway to decarbonisation in the building sector based on a policy review of major economies. *Advances in Climate Change Research* **16**, 183-198 (2025).

25. Y. Lan, J. Lu, S. Wang, An experimental study on the performance of TEGs using uniform flow distribution heat exchanger for low-grade thermal energy recovery. *Energy* **292**, 130506 (2024).

26. M. Al-Widyan, M. d. Al-Nimr, Q. Al-Oweiti, A hybrid TEG/Thermal radiator system for space heating and electric power generation. *Journal of Building Engineering* **41**, 102364 (2021).

27. K. Karthick, S. Suresh, G. C. Joy, R. Dhanuskodi, Experimental investigation of solar reversible power generation in Thermoelectric Generator (TEG) using thermal energy storage. *Energy for Sustainable Development* **48**, 107-114 (2019).

28. S. Mahmoud Al Shurafa, F. Basim Ismail, H. A. Kazem, T. Ee Sann, T. Abdel Hameed Almajali, Enhancing Photovoltaic-Thermoelectric Generator (PV-TEG) system performance via mathematical modeling and advanced thermal interface material: An emphasis on Pyrolytic graphite Sheet (PGS). *Solar Energy* **273**, 112514 (2024).

29. C. Liang *et al.*, Light-driven photothermal catalysis for degradation of toluene on CuO/TiO2 Composite: Dominating photocatalysis and auxiliary thermalcatalysis. *Applied Surface Science* **601**, 154144 (2022).

30. L. Huang, L. Xing, Y. Zheng, H. Yao, A chip thermal management method realizing integrated applications of cooling, power generation and heat flow measurement based on thermoelectric effect. *Applied Thermal Engineering* **258**, 124739 (2025).

31. T. Zhang *et al.*, Photothermal catalytic hydrogen production coupled with thermoelect ric waste heat utilization and thermal energy storage for continuous power generation. *Nano Energy* **121**, 109273 (2024).

32. T. Li, D. Wu, B. Li, X. Guo, A low-power thermoelectric power generation system based on a periodic thermoelectric power generation method in the form of heat pulses. *Energy* **308**, 132869 (2024).

33. B. Cao, Y. Tu, R. Wang, A Moisture-Penetrating Humidity Pump Directly Powered by One-Sun Illumination. *iScience* **15**, 502-513 (2019).

34. T. Li *et al.*, Simultaneous atmospheric water production and 24-hour power generation enabled by moisture-induced energy harvesting. *Nature Communications* **13**, 6771 (2022).

35. Q. Wu, W. Su, Q. Li, Y. Tao, H. Li, Enabling Continuous and Improved Solar-Driven Atmospheric Water Harvesting with Ti3C2-Incorporated Metal–Organic Framework Monoliths. *ACS applied materials & interfaces* **13**, 38906-38915 (2021).

36. Y. Zhang, D. Feng, X. Zhang, Y. Feng, Effect of paraffin wax with carbon nanotube on melting latent heat under different pressures: A molecular research. *Journal of Molecular Liquids* **382**, 121991 (2023).
